# Evaluating COVID-19 vaccination policy in Québec (Canada) using a data-driven dynamic transmission model

**DOI:** 10.1371/journal.pcbi.1013207

**Published:** 2025-08-25

**Authors:** Samuel Torres-Florez, Jorge Luis Flores Anato, Jiahuan Helen He, Vicente Garrido Portilla, Yichen Wu, Mathieu Maheu-Giroux, Étienne Racine, Caroline E. Wagner

**Affiliations:** 1 Department of Bioengineering, McGill University, Montréal, Québec, Canada; 2 Department of Epidemiology and Biostatistics, McGill University, Montréal, Québec, Canada; 3 Department of Physiology, McGill University, Montréal, Québec, Canada; 4 Welch Center for Prevention, Epidemiology, and Clinical Research, Johns Hopkins University, Baltimore, Maryland, United States of America; 5 Department of Epidemiology, Johns Hopkins Bloomberg School of Public Health, Baltimore, Maryland, United States of America; 6 Department of Chemical Engineering, University of Waterloo, Waterloo, Ontario, Canada; 7 Department of Electrical and Computer Engineering, McGill University, Montréal, Québec, Canada; 8 Institut national de santé publique du Québec, Québec City, Québec, Canada; Dartmouth College, UNITED STATES OF AMERICA

## Abstract

During the COVID-19 pandemic, decision-makers had imperfect information and faced resource constraints (i.e., vaccine availability). Public health decisions at the time may not have been optimal for minimizing disease burden. Here, we perform counterfactual evaluations of the impact of various vaccination strategies in Québec (Canada) on the COVID-19 burden from March 2020 to November 2021. In particular, we evaluate the effect of alternative age-specific prioritization sequences of vaccine dose roll-out and assess the impacts of vaccine hesitancy. To achieve this, we develop and calibrate a deterministic, compartmental dynamic transmission model, stratified according to age, susceptibility to infection, viral variant, and outcome-specific immunity. The initial conditions and parameters of the model are obtained through a combination of population-based surveillance data and Approximate Bayesian Computation Sequential Monte Carlo (ABC-SMC) parameter estimation methods. Using our calibrated model, we find that the vaccination prioritization policies implemented at the start of the pandemic, where age groups at highest risk were sequentially prioritized, was only outperformed by prioritizing the vaccination of younger, more socially connected groups together with higher-risk individuals aged 50+ (3% fewer hospitalizations compared to the baseline strategy). These results hold when we account for vaccine hesitancy. Specifically, we generally observe the fewest hospitalizations for the optimal strategies at the highest uptake rates (i.e. with the least vaccine refusal). However, certain sub-optimal strategies show higher hospitalization rates for higher vaccine uptake as a result of reduced vaccine dose redistribution to more interconnected age groups. Overall, our findings illustrate how the impact of vaccination strategies depends on population factors (e.g. contact patterns, vaccine uptake, and degree of immunity), age-specific risk of severe disease and transmission dynamics. Understanding these dependencies are important for guiding future decision-making related to priority vaccine administration in the face of known and emerging pathogens and potential shortages of doses.

## Introduction

The COVID-19 pandemic has presented extraordinary challenges to public health systems globally. This emergency has required quick and strategic decision-making in the absence of perfect and real-time information regarding the allocation of often limited resources. In many regions around the world, the initial shortage of vaccines forced public health authorities to prioritize vaccination among different demographics.

Québec is the second largest Canadian province by population and was the epicenter of that country’s initial epidemic in the spring of 2020 [1]. The first case in Québec was reported on February 28th 2020, leading to the province’s first public health emergency declaration shortly thereafter. Despite stringent physical distancing measures, Québec had the highest adjusted case fatality rate (CFR) among the most affected Canadian provinces in the first year of the pandemic, as well as the highest number of deaths among older individuals [2]. There were four waves of infection between spring 2020 and early winter 2021, driven by three main variants: wild-type, Alpha (B.1.1.7), and Delta (B.1.617.2) [3,4]. Vaccination in Québec began at the end of December 2020, initially prioritizing residents of long-term care homes (LTCHs) and healthcare workers (HCWs). By March 20, 2021, campaigns expanded to the general population, with the majority of doses being mRNA vaccines, such as Pfizer-BioNTech (BNT162b2) and Moderna (mRNA-1273) products. Due to limited vaccine availability, early distribution focused on HCWs, individuals with comorbidities, and older adults, before gradually expanding to younger age groups in descending order [5].

The optimal approach to vaccine allocation continues to be debated, leading to extensive research focused on optimizing vaccine allocation strategies across different diseases and demographic groups [6]. Previous influenza modeling research highlighted the strategic importance of targeting school-aged children and young adults in immunization campaigns to minimize infections and deaths due to their role in transmission and contact patterns [7]. During the H1N1 pandemic, modeling results showed that vaccinating adults aged 25-49 was consistently prioritized in optimal strategies, though the exact allocation strategy varied depending on vaccination timing and whether individual or population benefits were considered [8]. In the context of the COVID-19 pandemic, studies suggested that prioritizing vaccination for younger adults with more contacts from social and workplace interactions could effectively curb transmission rates [9] and deaths (when vaccine effectiveness is high) [10]. However, to achieve a significant reduction in mortality, modeling studies show that the vaccination of groups at higher risk of morbidity and mortality, particularly older adults for COVID-19, is likely the most effective strategy [11–16]. The success of vaccination programs is also strongly influenced by vaccine hesitancy, driven by factors such as misinformation, distrust in health and political authorities, and concerns about vaccine safety [17–21]. Therefore, it is critical to incorporate vaccine hesitancy into model-based policy evaluation efforts, as has been done in some models for the distribution of the COVID-19 vaccine [15,16], to ensure that projections accurately capture the uncertainty in achievable vaccine coverage due to reduced uptake arising from individual behavioral decisions.

Ultimately, the effectiveness of age-based vaccination strategies in mitigating infections, hospitalizations, and deaths for a given disease depends on factors related to disease natural history, population-level contact patterns, vaccine effectiveness and characteristics, and behavioral responses [22–27]. Evaluating historical vaccine prioritization schemes using dedicated modeling tools is a powerful way to study the relative importance of these variables to guide future public health decision making [6,28]. Here, we study COVID-19 hospitalizations in Québec prior to the emergence of the Omicron (BA.1) variant (i.e. up until November 2021) under historical and alternative age-based vaccination roll-out schemes, using a detailed age-stratified compartmental model, calibrated with empirical epidemiological data from Québec that accounts for specific population contact networks, distinct viral variants, vaccination and non-pharmaceutical interventions (NPIs), and the dynamics of immune waning and cross-protection. Specifically, we examine the impact of nine different vaccination schemes that differ via their initial age group prioritization and various degrees of population-level vaccine hesitancy on the projected time series for age-based hospitalizations.

## Methods

### Model structure

**Fig 1:**
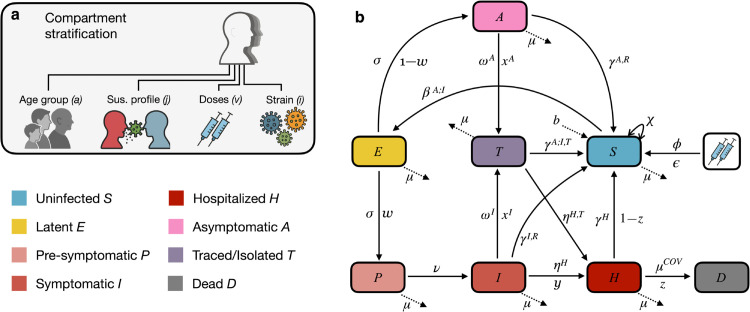
Schematic of stratification and inter-compartmental flows of the SARS-CoV-2 model of transmission and control. (**a**) Stratification of compartments by age, susceptibility to infection, number of vaccine doses received, and viral variants. (**b**) Model flow charts depicting transitions between compartments. *ϕ* is the number of first ($\phi_{1}$) and second ($\phi_{2}$) doses per unit time and 𝜖 the vaccine effectiveness. Dotted lines indicate demographic processes: *b* is the birth rate and *μ* the mortality rate. The transmission parameter, denoted by *β*, governs the infection dynamics for asymptomatic (*A*), presymptomatic (*P*), and symptomatic (*I*) individuals, with $\beta^{A}$ corresponding to the *A* and *P* compartments, and $\beta^{I}$ corresponding to the *I* compartment. *σ* is the inverse average latency period, *w* is the proportion of infected who become symptomatic (first going through a presymptomatic state, *P*), ν is the inverse average duration of the presymptomatic state, *ω* is the inverse average time to trace and isolate and *x* is the proportion infected symptomatic (*I*) or asymptomatic (*A*) individuals who are traced and isolated. $\gamma^{A;I,T}$ is the inverse average time asymptomatic individuals (*A*) or symptomatic individuals (*I*) spend in isolation before recovering, $\gamma^{A;I,R}$ is the inverse average time asymptomatic individuals (*A*) or symptomatic individuals (*I*) spend before recovering if they are not isolated, and $\gamma^{H}$ is the inverse average time to recovery in hospitalized patients. $\eta^{H}$ is the inverse average time between symptom onset and hospitalization and $\eta^{H,T}$ the inverse average time symptomatic individuals spend in isolation before being hospitalized. $\mu^{COV}$ is the inverse average duration of hospitalization before death, *z* is the proportion of hospitalized individuals who die from COVID-19, *y* is the proportion of infected individuals who are hospitalized, and χ is the inverse average duration of immunity. *All illustrative icons were created with the assistance of the GPT-4o image generation model from ChatGPT (OpenAI).*

The model stratifies the population by age, susceptibility to infection, vaccination status, and circulating viral variants (Fig 1a). The flow of individuals between these compartments occurs as follows. Uninfected individuals (S) can acquire infection through contact with infectious individuals (A for asymptomatic, P for presymptomatic, and I for symptomatic). Upon infection, individuals transition from S to E (latent infection), where they remain for an average duration equivalent to the disease’s latency period. After this period, they become infectious, either asymptomatic (moving from E to A) or presymptomatic (moving from E to P), depending on whether or not they will develop symptoms. Asymptomatic infectious individuals (A) follow one of two potential paths: they either recover directly and move back to S, or are traced and isolated, transitioning to T (traced) before recovering (back to S). Presymptomatic individuals (P) progress to symptomatic infection (I) after a brief period. From here, symptomatic individuals (I) follow one of four paths: (i) recovery without tracing or hospitalization (I to S), (ii) recovery post tracing and isolation but without hospitalization (I to T, then to S), (iii) hospitalization without prior tracing/isolation (I to H), or (iv) hospitalization following tracing and isolation (I to T, then to H). Hospitalized individuals (H) may either recover (H to S) or succumb to the disease (H to D). Upon recovery, individuals develop immunity (reduced susceptibility to infection). The schematic of these flows is shown in Fig 1b. In the results section, we present the values of model parameters pertaining to natural history of disease, both calibrated and extracted from the literature. The model is initialized by seeding infected individuals in the population according to surveillance data on the number of daily case importations extracted from Godin et al. [1]. For more information on model structure, see Section A–C in S1 Appendix. The differential equations describing the model are shown in Section F in S1 Appendix.

### Behavioral and policy responses

**Fig 2:**
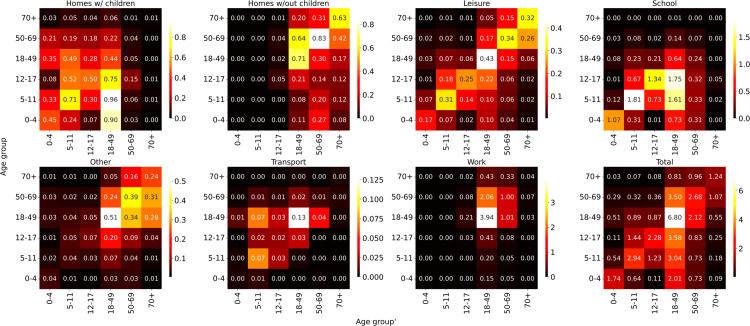
Model’s contact matrices. Number of contacts per day, per type by age group used in the model. Contact values are calculated by performing weighted averaging of the population sizes of the age groups considered in our model over the measured CONNECT matrices [29].

Our model is designed to simulate and analyze the impact of changes in population behavior and public health policies on the transmission dynamics of SARS-CoV-2. This includes the effects of measures such as social distancing and targeted vaccination efforts, which play a crucial role in altering the spread of the virus within the population. To model the impact of non-pharmaceutical interventions (NPIs) on virus transmission, we introduce reduction coefficients (κ) that multiply contact matrices representing interactions in different environments including homes, workplaces, transport, and leisure within and between age groups. These contact matrices are based on the pre-pandemic survey data reported in the CONNECT study, a population-based survey of social contacts in Québec [29], reduced to the age groups that are considered in the model as shown in Fig 2. The environment-specific κ coefficients reduce the number of effective contacts an individual has, reflecting the real-world effects of interventions like masking, lock-downs, school closures, and restrictions on gatherings (S5 Fig). Contacts are updated at specified time points, which are determined in part based on the timing of NPI policy implementation in Québec. For instance, schools were closed on March 16th 2020 and opened partially on May 11th 2020; the κ coefficient for school contacts is therefore set to zero during the closure period. The time series of calibrated κ values for all interaction types is shown in S5 Fig, and a detailed description of the implementation of NPIs in the model and their associated κ values is provided in Section E in S1 Appendix.

### Incorporation of SARS-CoV-2 variants

To account for the simultaneous circulation of different variants, we employ separate compartments for each variant, labeled by the index *i*. The model is structured to allow for any number of variants, in principle. However, we restrict our analysis to the wild-type, Alpha (B.1.1.7), and Delta (B.1.617.2) variants since they were dominant between March 2020 and November 2021. To accurately reflect the unique characteristics of each variant, the model assigns variant-specific values to disease natural history parameters, including transmissibility, incubation period, infectious period, and severity of outcomes associated with each variant. New variants are introduced into the model at specified time points, resulting in initial co-circulation and eventual dominance of the new variant. Additional details are provided in Section B and C in S1 Appendix.

### Modeling vaccination and hesitancy

We assume that vaccination is exclusively administered to individuals within the uninfected (S) compartment, following the government guidelines that exposed/infected individuals should not seek vaccination. Furthermore, the prevalence of infection was low, around 3%, when vaccination started (March 2021) [30], suggesting that error arising from this assumption should be minimal. Vaccination of the general population began in March 2021 with BNT162b2 (Pfizer–BioNTech), mRNA-1273 (Moderna), or ChAdOx1 CoV-19 (Oxford–AstraZeneca). For this study and consistency with the simulated time frame (March 2020–November 2021), we consider a simplified vaccination schedule of at most two mRNA doses. Hence, we define three distinct strata: unvaccinated (*v* = 0), vaccinated with the first dose (*v* = 1), and vaccinated with two doses (*v* = 2).

We account for the variability in vaccine effectiveness against different virus variants by offering protection against infection and hospitalization. Protection against infection conferred by the vaccine, for each variant *i*, is modeled by an efficacy matrix. Upon administration of a new vaccine dose, an individual’s initial susceptibility profile $j^{\prime }$ transitions to a final profile *j* based on the efficacy of the vaccine dose *v* against infection caused by variant *i* in a specific age group *a*. For further details on vaccination and its effect on outcomes, refer to Section B in S1 Appendix. Protection against hospitalization is modeled as an age- and variant-specific, population-level value. The probability of being hospitalized following infection (denoted as *y*_*i*_) is stratified according to vaccine status *v*. This approach allows us to account for risk and protective factors in an age-specific manner within the population.

**Fig 3:**
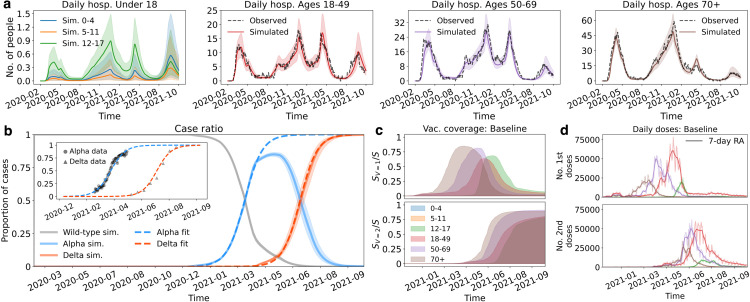
Calibrated model outputs. (**a**) Simulated (fitted) curves for daily hospitalizations; ages are color coded and the dashed lines represent the observed hospitalizations as a 7-day rolling average. Note that the observed hospitalizations are not shown in the plot corresponding to individuals <18 (see the text for additional details). The shaded regions indicate the 95% credible interval. (**b**) Proportion of cases attributable to each variant. The inset plot illustrates a logistic fit applied to raw data representing the proportions of cases screened positive for the Alpha and Delta variants. The logistic fit served as the calibration target rather than the raw data for efficiency reasons. The main graph shows the dynamics of the wild-type, Alpha, and Delta variants during the simulated time period. (**c**) The proportion of the (uninfected) population that have received one ($S_{v=1}/S$) or two ($S_{v=2}/S$) doses over time. (**d**) Number of daily doses administered by age group. The colours correspond to the age groups in (**c**).

To simulate the administration of vaccine doses, the model calculates the daily number of eligible uninfected individuals for either the first or second dose. Then, the number of doses administered on a given day within this identified population is determined based on observed vaccine coverage data (obtained from the institut national de santé publique du Québec (INSPQ) [31]), ensuring that the number of daily doses administered is consistent across scenarios and based on actual vaccine availability at that time. Additional details are provided in Section D in S1 Appendix. Observed vaccination coverage by age and number of doses is shown in Fig 3c. In our baseline scenario, we follow the age-based roll-out calendar adopted for the general public in Québec, initially focusing on the 70+ age group and, subsequently, other age groups in descending order. Fig 3d further details the roll-out of first and second doses. The proportion of each age group that received one or two doses in the considered time frame is showed in S1 Table.

We consider scenarios where full coverage could be achieved and scenarios where vaccine hesitancy is considered. In all scenarios, once all eligible individuals in the priority age group have been vaccinated, the remaining available doses on a given day are distributed uniformly among the remaining age groups (i.e., the remaining doses are divided equally between the remaining age groups). For the second dose, the same age-based prioritization scheme is followed as for the first dose by scenario, except the timing of administration of the second dose is set to coincide with the historical roll-out. To integrate the concept of vaccine hesitancy, we impose a cap on maximum vaccine coverage, reflecting the reality that not all individuals will accept vaccination. For each hesitancy scenario, we assume a uniform cap in vaccine uptake across all age categories except for the 70+ cohort. Due to the high recorded vaccine uptake in this age group in Québec [32], we assume that all susceptible individuals in this age group accept vaccination. For example, to model a population where 30% of the individuals are reluctant to vaccinate, we set a maximum coverage of 70% in all age groups other than the 70+ cohort. This means that when this maximum coverage is achieved in a specific age group, we will stop distributing doses to that cohort and continue vaccinating groups where the maximum coverage has not yet been achieved according to the priority scheme in question.

### Cross-protection and immunity

Cross-protection occurs when recovery from infection by one variant confers some protection against subsequent infections by different variants. Cross-protection is modeled by stratifying individuals by their infection status and immunity levels against each circulating variant. For instance, an individual who has recovered from an infection by the Alpha variant may exhibit reduced susceptibility to the Delta variant. This reduced susceptibility (acquired immunity) is modeled through the susceptibility profile indices *j*. See Section C in S1 Appendix for details on how cross-protection is implemented in the model.

### Waning immunity

Waning immunity is a critical factor that can influence the trajectory of a pandemic and the effectiveness of vaccination programs. Previous studies on antibody-mediated immunity to SARS-CoV-2 and other coronaviruses such as SARS-CoV, MERS-CoV, and the four endemic human coronaviruses (HCoVs) estimated that infection-derived protection against SARS-CoV-2 is likely to last for 1-2 years, suggesting similar dynamics for vaccine-induced antibodies [33]. Our model incorporates waning immunity, which occurs following natural infection, vaccination, or a combination of both. We assume that waning of any immunity, whether acquired from natural infection or vaccination, is not variant-specific. This means that waning concurrently affects immunity to all circulating variants. Also, we only consider waning immunity against infection and assume that vaccine-induced immunity against hospitalizations is permanent, a reasonable assumption given our short modeling time horizon. For details on how waning immunity is implemented, see Section C and D in S1 Appendix.

### Calibration

Table 1 lists all calibrated model parameters. These parameters were calibrated by fitting the model predictions to the observed proportion of cases attributed to the different variants, obtained from screenings of specific mutations (N501Y, del69/70, and E484K) [34] as well as daily new hospitalizations by age over time, obtained from the *Maintenance et exploitation des données pour l’étude de la clientèle hospitalière database (MED-ÉCHO live)* [35], filtering presumed nosocomial infections as well as transfers from long-term-care homes (LTCHs), see S2 Fig. Severe cases in the eldest age groups in Québec during the first wave were often due to infections in LTCHs [36,37]. Since the transmission dynamics in LTCHs are distinct from those of the general population, we remove hospitalizations arising from LTCHs from our observed hospitalization data for calibration. Calibration was performed using an Approximate Bayesian Computation - Sequential Monte Carlo (ABC-SMC) algorithm [38] in which parameter posterior distributions could be estimated without the need for explicit likelihood functions, using summary statistics and distance metrics to measure the fit between model outputs and observed data. The priors were defined as beta distributions whose mode was determined by calibrating the model by hand and based on measured values from the literature. The variance of each beta distribution was determined heuristically to achieve a sufficient acceptance rate during calibration. For an in-depth overview of the calibration methodology, including the calibrated parameters, specific targets, and metrics used, see Section G in S1 Appendix.

**Table 1:** Calibrated parameters. Parameters chosen for calibration based on their influence on model dynamics and their level of uncertainty or variability in the literature.

Symbol	Definition
$\rho_{i}^{I}$	Baseline probability of transmission for symptomatic infectious individuals per contact by variant.
$\rho_{i}^{A}$	Baseline probability of transmission for asymptomatic infectious individuals per contact by variant.
*y*_*a*_	Proportion of infected individuals that will be hospitalized by age group.
*w*_*a*_	Proportion of infected individuals that will develop symptoms by age group.
$x_{a,i}^{A}$	Proportion of infectious asymptomatic individuals that will be traced and isolated by age group and variant.
$x_{a,i}^{I}$	Proportion of infectious symptomatic individuals that will be traced and isolated by age group and variant.
$I_{\alpha_{0}}$	Proportion of seeding cases attributed to the alpha variant.
$I_{\delta_{0}}$	Proportion of seeding cases attributed to the delta variant.
κ	Reduction coefficients of the number of contacts per unit time.

### Vaccine effectiveness against infection, hospitalization and death

Vaccine effectiveness values (Table 2) are obtained from test-negative design studies performed in Canada, specifically focusing on mRNA vaccines. The vaccine effectiveness values against wild-type and Alpha infection after one and two doses are extracted from a study among healthcare workers in Québec by Carazo et al. [39]. One-dose vaccine effectiveness values against wild-type, Alpha, and Delta hospitalization, as well as Delta infection are obtained from Ontario studies by Nasreen et al. [40]. Two-dose vaccine effectiveness values against wild-type, Alpha, and Delta hospitalization as well as Delta infection are obtained from British Columbia and Québec studies by Skowronski et al. [41]. For all age groups, including those aged 70 and above, the two-dose vaccine effectiveness is assumed to be the same. However, for the 70+ age group, a 3% relative reduction in vaccine effectiveness is applied for single-dose protection [39].

**Table 2:** Vaccination effectiveness. Effectiveness against infection, hospitalization, and death by age and number of doses.

Effectiveness against	Age	Wild-type		Alpha		Delta	
		1${\;\;}^{\text{st}}$ dose	2${\;\;}^{\text{nd}}$ dose	1${\;\;}^{\text{st}}$ dose	2${\;\;}^{\text{nd}}$ dose	1${\;\;}^{\text{st}}$ dose	2${\;\;}^{\text{nd}}$ dose
Infection	0-69	77.00 ${\;\;}^{◁}$	86.50 ${\;\;}^{◁}$	60.00 ${\;\;}^{◁}$	92.60 ${\;\;}^{◁}$	64.00 ${\;\;}^{⋄}$	88.00 ${\;\;}^{▷}$
	70+ ^*^	74.70 ${\;\;}^{◁}$	86.50 ${\;\;}^{◁}$	58.20 ${\;\;}^{◁}$	92.60 ${\;\;}^{◁}$	62.10 ${\;\;}^{⋄}$	88.00 ${\;\;}^{▷}$
Hosp. & death	0-69	88.00 ${\;\;}^{⋄}$	98.00 ${\;\;}^{▷}$	84.50 ${\;\;}^{⋄}$	98.00 ${\;\;}^{▷}$	86.00 ${\;\;}^{⋄}$	97.00 ${\;\;}^{▷}$
	70+ ^*^	85.40 ${\;\;}^{⋄}$	98.00 ${\;\;}^{▷}$	82.00 ${\;\;}^{⋄}$	98.00 ${\;\;}^{▷}$	83.40 ${\;\;}^{⋄}$	97.00 ${\;\;}^{▷}$

◁ values obtained from Carazo et al. [39], ⋄ values obtained from Nasreen et al. [40], and ▷ values obtained from Skowronski et al. [41]. * 3% relative reduction for single-dose vaccine effectiveness in 70+ age group.

### Model parameters

Various sources were used to inform the age-stratified model parameters shown in Table 3. The birth rate value (*b*) was extracted from Québec Statistics data [42] and the value represents 9.5 births per 1000 people/year. The average latency period (1/σ) and the duration of the pre-symptomatic state (1/ν) were determined based on studies of COVID-19’s incubation period and early infectiousness [43–48]. The average time to trace and isolate an infected individual (1/ω) relies on expert opinion due to variability in contact tracing efficiency. Hospitalization-related parameters, such as the average time to being hospitalized ($1/\eta^{H}$) and the duration of hospitalization before death ($1/\mu^{COV}$), were sourced from statistical studies on COVID-19 hospitalizations [49]; the values for the proportion of hospitalized individuals who die from COVID-19 (for the wild-type variant and without vaccination, $z_{v=0}$) are extracted from in-hospital mortality risk studies in Québec [50]. The average disease duration ($1/\gamma^{I,R}$) and recovery time for hospitalized patients ($1/\gamma^{H}$) were derived from research on the infectiousness of SARS-CoV-2 and patient recovery trajectories [50–53]. The all-cause mortality rate (*μ*) is based on data from Statistics Canada [54], representing rates per 1000 people/year. The timescale for immunity waning (1/χ) was based on values from previous coronaviruses [33] and fitted in sensitivity analyses.

**Table 3:** Model parameters. Calibrated values are identified by † and show the [95% CI].

	Definition	0-4	5-11	12-17	18-49	50-69	70+	Ref.
*b*	Birth rate ${\;\;}^{⋆}$	9.50/*y*	0	0	0	0	0	[42]
1/σ	Average latency period ${\;\;}^{⊖}$	4.60*d*						[43,44]
1/ν	Average duration of presymptomatic state ${\;\;}^{⊖}$	1.80*d*						[45–48]
1/ω	Average time to trace and isolate ${\;\;}^{⊖}$	5.50*d*						Expert Op.
$1/\eta^{H}$	Average time to being hospitalized	1.45*d*	1.45*d*	3.35*d*	2.75*d*	2.75*d*	1.80*d*	[49]
$1/\gamma^{I,R}$	Average duration of disease ${\;\;}^{⊖}$	8.50*d*						[51,52]
$1/\gamma^{H}$	Average time to recovery after being hospitalized	3.40*d*	3.70*d*	5.50*d*	6.80*d*	12.20*d*	18.60*d*	[50,53]
$1/\mu^{COV}$	Average duration of hospitalization before death	9.20*d*	9.20*d*	9.20*d*	9.20*d*	12.30*d*	9.60*d*	[49]
*μ*	All-cause mortality rate ${\;\;}^{⋆}$	0.90/*y*	0.10/*y*	0.15/*y*	0.61/*y*	5.65/*y*	45.45/*y*	[54]
1/χ	Average duration of immunity ${\;\;}^{⊖}$	730*d*						S. A
*x* ${\;\;}^{\text{I}}$	Proportion of infected symptomatic individuals who are traced and isolated ${\;\;}^{⊖}$	0.62 [0.57, 0.66]						†
*x* ${\;\;}^{\text{A}}$	Proportion of infected asymptomatic individuals who are traced and isolated ${\;\;}^{⊖}$	0.56 [0.49, 0.64]						†
*w*	Proportion of infected individuals who become symptomatic	0.60 [0.55, 0.67]	0.46 [0.42, 0.51]	0.17 [0.12, 0.21]	0.18 [0.15, 0.20]	0.31 [0.22, 0.36]	0.83 [0.74, 0.85]	†
$y_{v=0}$	Proportion of symptomatic, unvaccinated infected individuals who are hospitalized	0.01 [0.00, 0.02]	0.00 [0.00, 0.01]	0.05 [0.02, 0.08]	0.02 [0.02, 0.03]	0.07 [0.06, 0.11]	0.23 [0.20, 0.26]	†
$z_{v=0}$	Proportion of hospitalized individuals who die from COVID-19	0	0	0.018	0.044	0.16	0.51	[50]

⊖ = age-independent, † = calibrated parameter, Expert Op. = expert opinion, S. A = sensitivity analysis.

⋆ = birth and mortality rates per 1000 people/year.

## Results

### Model fitting and predictive performance

#### Parameter estimation.

The complete list of model parameters and their values is provided in Tables 2–4. The estimated values for the contact reduction coefficients are shown in S5 Fig. Table 3 shows parameter values obtained from (i) the literature, (ii) expert opinion, (iii) sensitivity analysis, and (iv) fitting (calibration). Fitted parameter values (indicated by a †) were obtained by calibrating the model to daily hospitalizations by age and to the fraction of infections attributable to each variant (see Section G in S1 Appendix for additional details). It is important to note that the model calibration process did not involve fitting to case incidence data.

**Table 4:** Transmission parameters by variant. Calibrated values for $\rho_{i}^{I}$ and $\rho_{i}^{A}$ show the [95% CI].

	Probability	Wild-type	Alpha	Delta
$\rho_{i}^{I}$	Baseline probability of transmission for symptomatic infectious individuals per contact by variant${\;\;}^{†}$	0.06 [0.06, 0.06]	0.16 [0.14, 0.17]	0.34 [0.31, 0.37]
$\rho_{i}^{A}$	Baseline probability of transmission for asymptomatic infectious individuals per contact by variant${\;\;}^{†}$	0.78 [0.75, 0.81]	0.53 [0.47, 0.62]	0.46 [0.39, 0.52]

† = calibrated parameter.

Table 4 presents the calibrated values for (i) the baseline probabilities of transmission for symptomatic infectious individuals per contact by variant ($\rho_{i}^{I}$), and (ii) the baseline probabilities of transmission for asymptomatic infectious individuals per contact by variant ($\rho_{i}^{A}$). For details on transmission rates, see Section B in S1 Appendix. Model calibration indicates an increasing baseline probability of transmission for symptomatic infectious individuals for each new variant, suggesting that Delta was 2.13 times more transmissible than Alpha. This aligns with previous research that possibly attributes Delta’s enhanced transmissibility to its ability to generate around 6-times more viral RNA copies per mL than Alpha infections [55]. Additionally, we find that the transmission probability for asymptomatic individuals is highest for the wild-type variant ($\rho_{\text{WT}}^{A}=0.78$), followed by the Alpha ($\rho_{\text{Alpha}}^{A}=0.53$) and Delta ($\rho_{\text{Delta}}^{A}=0.46$) variants. The detailed prior distributions associated with each fitted parameter are shown in S6 Fig.

#### Daily hospitalizations, variant emergence, and vaccine roll-out.

The model’s predicted hospitalization time series post-calibration are shown in Fig 3a, where the predicted daily hospitalizations and actual hospitalization incidence are plotted by age group. We combined ages below 18 in a single plot (the left panel of Fig 3a) because the low frequency of hospitalizations in children created highly variable (noisy) data, making it challenging to fit our deterministic model to these age groups. Overall, however, the magnitude of hospitalizations is well reproduced (a more detailed overview of these age groups is shown in S3 Fig). For individuals aged 18 and over, excellent agreement is observed between the simulated hospitalizations (fitted outputs) and historical data in all age groups. As shown in Fig 3b, the model also accurately simulates the temporal dynamics of variant replacement.

### Assessment of vaccine allocation strategies

**Fig 4:**
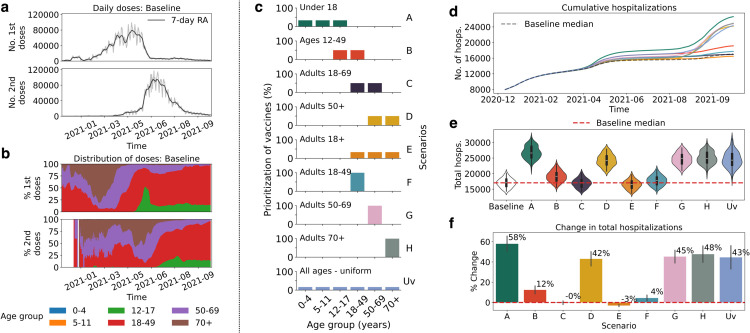
Outcomes of vaccine allocation strategies on hospitalizations. (**a**) Total number of first (top) and second (bottom) doses administered each day. (**b**) Proportion of first (top) and second (bottom) doses administered daily to each age group in the baseline scenario. (**c**) Percentage of doses prioritized for each age group at the start of vaccination for nine scenarios. A: Under 18, B: Ages 12-49, C: Adults 18-69, D: Adults 50+, E: Adults 18+, F: Adults 18-49, G: Adults 50-69, H: Adults 70+, and Uv: All ages. (**d**) Cumulative hospitalization counts from mid-December 2020 to November 2021. The dashed line shows the median values from the baseline scenario and the solid lines are the median values for each scenario. (**e**) Total hospitalizations over the simulated time-period, for the nine proposed scenarios and the baseline scenario (dashed red line). (**f**) Percent change in total hospitalizations for the nine proposed scenarios relative to the median value from the baseline scenario. The color scheme in all plots corresponds to that in (c).

Our primary objective was to evaluate how alternate vaccination strategies may have impacted hospitalization time series, focusing specifically on age-based prioritization schemes when vaccine doses were initially in limited supply. The total number of doses administered each day is shown in Fig 4a. Fig 4b shows the proportion of daily doses that are administered to each age group in the baseline scenario. The age-based strategies considered are shown in Fig 4c.

We tested vaccine allocation strategies by prioritizing sets of three age groups (e.g., three groups representing children under 18: 0–4, 5–11, 12–17; or three adult age groups: 18–49, 50–69, and 70+), pairs of adjacent age groups (e.g., ages 12-49), and targeted individual age groups (e.g., adults 18-49). For clarity, each scenario is identified using a letter from A-H. The uniform vaccination strategy (identified as Uv) is designed to divide doses equally among all age brackets until the number of eligible individuals within an age group has been reached (i.e., no one eligible is left to vaccinate). The number of daily doses remains equal and is therefore consistent across all scenarios; only the age-specific vaccine coverage is modified. For detailed views of the evolution of the proportion of doses administered per age group in all simulated scenarios, see S8 Fig.

Fig 4d tracks cumulative hospitalizations across scenarios, with baseline scenario median values indicated by a dashed line. Strategy A, focusing on individuals under 18, initially experiences a higher rate of hospitalizations around May 2021, with strategies D (adults 50+), G (adults 50-69), H (adults 70+), and Uv (uniform) following a similar pattern but with lower magnitude. We also see a sharp increase in hospitalizations for these strategies in mid-August 2021, due to the arrival of Delta. Conversely, strategy B, which targets ages between 12 and 49, results in increased hospitalizations but at a lower rate compared to strategies D, G, H and Uv. Strategy F, which targets 18-49 adults, results in marginally higher hospitalizations compared to baseline.

In Fig 4e, we plot the total number of hospitalizations over the entire time window considered for each strategy, again comparing to baseline median values (the dashed red curve). Strategies targeting ages 18-69 (C), 18+ (E), and 18-49 (F) result in total hospitalizations comparable to the baseline. In Fig 4f, we plot the percent change in total hospitalizations across all scenarios compared to the baseline strategy. Vaccinating individuals under 18 years of age as a priority leads to a 58% increase in hospitalizations, while focusing on the oldest adults (70+) results in a 48% increase in total hospitalizations. Strategies G (50-69) and D (50+) also show elevated hospitalization rates, at 45% and 42% increases, respectively. Strategy Uv shows a ∼43% increase. Vaccination strategy B (12-49) results in a 12% increase. In contrast, strategies prioritizing ages 18-69 (C), adults 18+ (E), and adults 18-49 (F) approach baseline hospitalizations, differing by 0%, –3%, and 4%, respectively. Hence, the highest reductions in hospitalizations are seen when young adults with high contact rates are targeted jointly with high-risk (older) age groups.

### Temporal analysis of vaccine prioritization strategies

**Fig 5:**
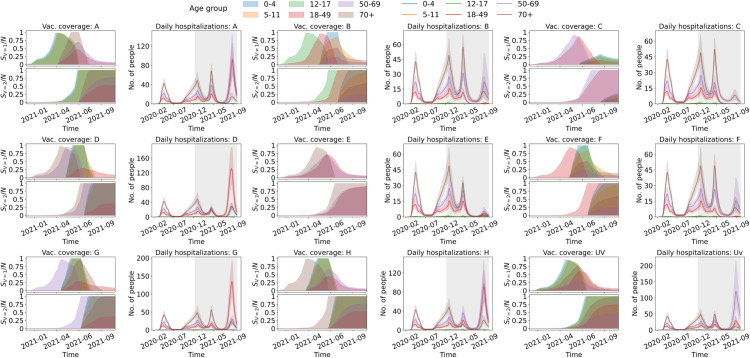
Effect of vaccine prioritization on hospitalization dynamics. Vaccination coverage (left) and daily hospitalizations (right) for each of the nine vaccination scenarios simulated. The shaded regions in daily hospitalization plots correspond to time periods where vaccines are being distributed within the population.

Fig 5 shows the evolution of vaccination coverage and daily hospitalizations by age group across the different scenarios. Coverage peaks and timing reflect both the age-based prioritization schemes of the different scenarios as well as the population size of each age group. For instance, scenario A, which prioritizes the under-18 cohort, demonstrates approximately synchronous coverage peaks across subgroups 0-4, 5-11, and 12-17. In contrast, scenario B, prioritizing ages 12-49, reveals a staggered achievement of coverage, with delayed rates of coverage in the 18-49 group—the largest and more interconnected demographic (see Figs 2 and S1 Fig). Notably, some scenarios, such as E, which focuses on adults 18+, result in incomplete coverage for non-prioritized groups, with the youngest cohorts receiving no vaccination.

S7 Fig and S8 Fig show a detailed chronological analysis of vaccine distribution by dose across the different age groups. Here, the influence of age group size on vaccine allocation is more clearly seen. For example, the number of doses necessary to vaccinate all individuals in the under-18 cohort (as is the objective of scenario A) is markedly lower than those required to vaccinate all adults (the 18+ age groups, i.e., the objective of scenario E). This implies an earlier redistribution of vaccines to non-priority age groups when smaller-sized priority groups are targeted. We can see this behavior when comparing the timing of the coverage peaks for the first doses on the non-priority groups in scenarios A and F in Fig 5.

Fig 5 shows disparities in hospitalization dynamics that underscore the distinct impacts of each vaccination strategy on different age demographics. In particular, scenarios A (under 18), D (50+), G (50-69), and H (70+) result in substantially higher hospitalization peaks, notably during the fourth wave driven by the Delta variant, representing an acute burden on the healthcare system. The plots in Fig 5 show that these peaks are mainly driven by hospitalizations among the 18-49 and 50-69 age cohorts, and less so by individuals aged 70+. This is because the aforementioned scenarios do not prioritize the 18-49 age group for vaccination, which leads to elevated rates of transmission. The uniform vaccination strategy (Uv) also results in a prominent Delta wave, primarily due to hospitalizations among the 50-69 age group. In contrast, strategies targeting the 18-69 groups (C), as well as all adults (E) and the 18-49 cohort (F), are associated with lower hospitalization peaks, indicating a mitigating effect on severe disease incidence during the Delta wave compared to other simulated strategies. Overall, scenarios that target both highly connected individuals (18-49) and high-risk adults (50+) show lower hospitalization burden approaching the baseline results.

### Vaccine hesitancy

**Fig 6:**
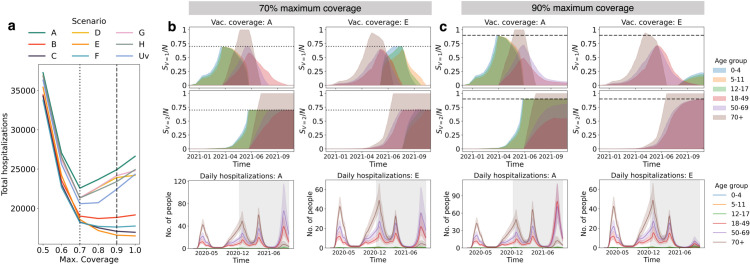
Effect of vaccination hesitancy on total hospitalizations. (**a**) Number of total hospitalizations as a function of maximum coverage from 40% to 100%. (**b** and **c**) Temporal dynamics of vaccination coverage (top) and daily hospitalizations (bottom) for 70% maximum coverage (b) and 90% maximum coverage (c) in scenarios A (under 18) and E (Adults 18+).

Overcoming vaccine hesitancy is a major obstacle in achieving collective immunity against a highly transmissible virus. In Fig 6a, we explore the impact of vaccine hesitancy on overall hospitalizations across vaccination scenarios, modifying the observed achieved proportion of immunized individuals with one or two doses (see S1 Table). Scenarios that prioritize the 18-49 age group, notably B (12-49), C (18-69), E (18+), and F (18-49), generally feature increasingly low levels of hospitalization with decreasing levels of hesitancy (or increasing maximum coverage). In contrast, strategies A (under 18), D (50+), G (50-69), H (70+), and Uv (uniform vaccination) show a rise in hospitalizations beyond 70% coverage thresholds although a slight decline at 100% coverage is observed in scenarios D and G. The behavior in scenarios B, C, E, and F is a consequence of the overall transmission reduction when the highly socially connected 18-49 age group is prioritized for vaccination. When this cohort is not prioritized, high vaccine uptake levels in other age groups results in fewer doses being redistributed to the 18-49 group, leading to higher transmission and hospitalizations within the population. Essentially, earlier saturation of vaccine coverage in prioritized age groups due to hesitancy allows for more timely reallocation of limited doses towards the 18-49 group. Interestingly, in scenarios B and F, where the 18-49 age group is targeted but those aged 50-69 are not, we observe a plateau and a slight increase in hospitalizations at coverage rates exceeding 90%. This subtle trend likely occurs because, although the 18-49 age group is vaccinated, the lack of redistribution to the 50-69 age group leads to increased hospitalizations in this older cohort. However, it’s important to note that the strategies represented by scenarios C and E, which target both the 18-49 and older age groups, demonstrate a consistent, monotonic trend in reducing hospitalizations, making them the overall optimal strategies.

To illustrate this, in Fig 6b-c we plot vaccination coverage over time (top two panels) and daily hospitalizations (bottom panel) for scenarios A (prioritization of individuals under 18) and E (prioritization of ages 18+) assuming maximum coverage levels of 70% (Fig 6b) and 90% (Fig 6c). For scenario E, decreases in vaccine hesitancy allow for higher coverage rates in the 18-49 and higher-risk (50+) age groups prioritized for vaccination, resulting in decreases in hospitalization across all adult age groups. We see this by comparing the magnitude of the fourth wave (attributed to the Delta variant) for scenario E in Fig 6b and 6c. In contrast, when individuals under 18 are prioritized (scenario A), an increase in hospitalizations during the Delta wave is observed for lower degrees of vaccine hesitancy. This increase in hospitalization is primarily attributed to cases in the 18-49 age group, which is less vaccinated at lower hesitancy due to reduced and delayed vaccine redistribution. Despite the redistribution of vaccines to ages 18+ after vaccinating individuals under 18, the delay in targeting adults results in overall higher hospitalizations, likely due to higher transmission levels in the 18+ age groups.

Detailed plots outlining the vaccination coverage and hospitalization data for all scenarios and maximum vaccine coverage levels of 50%, 70%, and 90% are shown in S10 Fig. We find that when vaccine uptake is low (50%), vaccination has minimal impacts on transmission dynamics, and high levels of hospitalizations during the fourth (Delta) wave are observed across scenarios. Indeed, with high hesitancy, even scenarios that prioritize the highest risk 70+ age group (for which maximum coverage is assumed to remain 100%) result in high levels of hospitalization, including among individuals 70+, due to elevated population-level transmission. When vaccine uptake is high, prioritizing younger adults (18-49) with more social contacts combined with prioritization of adults aged 50+ contributes to a reduction in hospitalizations, particularly during the fourth (Delta) wave. However, strategies not targeting the 18-49 cohort see higher overall hospitalizations due to more sustained population-level transmission. S11 Fig and S12 Fig illustrates the impact of vaccine hesitancy on the total number of doses administered. As hesitancy increases, the total number of doses administered to the population naturally decreases. Importantly though, for a given coverage level, the total number of doses remains nearly constant across all scenarios.

## Discussion

Overall, we find that the vaccination prioritization policies established at the start of the pandemic were essentially optimal compared to the age-stratified strategies that we consider in this study. However, we highlight the importance of prioritizing individuals with large numbers of contacts—such as the highly connected (i.e., highly mixed with other age groups and high number of contacts) 18-49 cohort in our model—when aiming to reduce hospitalization burden. These outcomes are consistent with prior research conducted in Canada, which indicated that immunizing younger individuals contributed to a reduction in cases of severe disease during the Delta wave [56,57]. Furthermore, our results agree with studies emphasizing the significance of vaccinating highly connected, socially active demographics [9]. In addition, economically active demographics have shown to be more likely to acquire (and transmit) the infection in the workplace and public spaces [58,59]. Nonetheless, the greatest reductions in hospitalizations occur when strategies target the 18-49 cohort in combination with adults aged 50+, who are at higher risk of morbidity and mortality. Our analysis highlights the critical nature of timing in vaccination campaigns. Specifically, although all the 18+ age groups eventually received vaccines via reallocation in all scenarios where they were not prioritized, the associated temporal delay and reduction in coverage relative to the timing of the arrival of the Delta variant resulted in high levels of hospitalizations during the fourth (Delta) wave.

Moreover, our study highlights the complex interplay between vaccine hesitancy and the efficacy of vaccination strategies in achieving population-wide protection. Recognizing that a significant portion of the population, particularly younger age groups, may be reluctant to vaccinate [60,61], we included vaccination hesitancy as vaccine coverage caps in all age groups other than the oldest adults (70+). We find that when maximum coverage is high (less than 30% hesitancy), strategies that do not prioritize the 18-49 age group may see increasing hospitalizations with increasing vaccine uptake due to reduced vaccine reallocation to this age group and correspondingly more sustained levels of disease transmission. However, even the minimum hospitalization levels for these non-optimal strategies (observed at around 30% hesitancy) are still higher than those seen in scenarios with high uptake in the optimal strategies. This underscores the fact that a poor strategy, coupled with hesitancy, is not a viable alternative and may lead to higher disease burdens. Given the inherent unpredictability of emergence of new variants and ensuing waves of infection, aiming for maximum achievable coverage as rapidly as possible likely remains the optimal approach for minimizing overall disease burden. It also highlights the importance of developing prioritization strategies robust to uncertainties in uptake levels. Proactive efforts to address hesitancy through evidence-based communication remain necessary for successful vaccination campaigns [62–65].

### Limitations and caveats

Some limitations and caveats should be considered when interpreting these results. In this study, we use two main data sources as targets for calibrations: (i) daily new hospitalizations by age group and (ii) the proportion of cases attributed to the Alpha and Delta variants obtained from screening for specific mutations. This focus inherently restricts our analysis to hospitalization outcomes. Yet, previous work using mathematical models [7] has shown that for diseases with age-based health outcomes, optimal vaccination strategies may vary depending on the targeted outcome measure (i.e. hospitalizations or deaths). Here, our focus was on identifying vaccination strategies that would minimize the overall burden on public healthcare systems generated by hospitalizations. Although we excluded LTCH data, where a high proportion of deaths occurred, the robustness of our hospitalization risk estimates indicates that the model still reliably captures the broader case trends. However, our analysis is limited by the lack of calibration to reliable case or seroprevalence data, which would have improved the quantitative interpretability of parameter estimates. Additionally, we lack insight into which parameter combinations the model’s equations are most sensitive to, potentially affecting our understanding of parameter interdependencies. In both cases, the availability of more diverse sources of high-quality data (such as serological surveillance data as proxies for case numbers) would improve the quantitative interpretability of our parameter estimates. Also, our pre-pandemic contact matrices are subject to biases inherent to the CONNECT study [29]. While we partially mitigated this issue by calibrating contacts during the pandemic, it remains possible that the overall age structure of contacts in our model may not provide an accurate representation of reality.

Additionally, we do not capture the effects of the vaccine passport implemented in Québec (September 2021) for leisure activities, which may have impacted contact rates, particularly among younger age groups [66]. Further, HCWs were also targets of early vaccine roll-out, which would be factored into the baseline scenario through our use of the historical vaccine dose schedule. In our hypothetical scenarios, however, we do not account for this group separately in terms of receiving early vaccination. In addition, using a uniform immune waning rate across all age groups, for all variants, and for all origins of immunity (i.e., vaccine or infection-derived) is a simplification. Indeed, hybrid immunity is not considered in our model although there is evidence that it may increase overall vaccine effectiveness and protection against infection [67–69]. Ultimately, the impact of non-uniform waning and hybrid immunity is less critical to this work since we only consider the pre-Omicron era in which cumulative incidence of infection remained fairly low and vaccine effectiveness against infection remained relatively high over time. However, future studies that include Omicron waves should consider these factors to model waning of immunity more realistically. For the same reason, our omission of the Omicron wave from this study also limits our ability to fully assess the efficacy of different vaccination strategies, as vaccine-induced immunity was particularly relevant when the Omicron variant was dominant and infection rates drastically increased. Finally, we emphasize that reducing hospitalizations by vaccinating the highly connected 18-49 age group relies on high vaccine effectiveness against infection by pre-Omicron variants. Similar results would likely not be obtained during the Omicron wave because vaccine effectiveness against infection was much lower for this variant and its sublineages.

## Conclusion

This work presents a highly stratified dynamic transmission compartmental model for COVID-19 in Québec that accurately reproduces observed hospitalization incidence by age and prevalence of variants over time before the introduction of the Omicron variant. By studying alternate vaccination roll-out strategies, we find that the immunization policy proposed at the start of the pandemic was nearly optimal in reducing hospitalization numbers compared to the alternative scenarios considered in this work. However, our results underscore that targeting broad and highly connected age groups (particularly the 18-49 age group considered here) could contribute significantly to reducing population-wide levels of hospitalization. Our analysis emphasized the significance of considering social connectivity in vaccine roll-out plans, alongside age and vulnerability, to reduce hospitalization incidence effectively. Additionally, our analysis highlights the critical role of vaccine hesitancy in shaping the success of vaccination campaigns.

Our model provides a robust platform for simulating the interplay between vaccination strategies, disease dynamics, and social behaviors. Additionally, by altering the disease’s natural history, the model can be adapted to describe the transmission of other pathogens. In the context of COVID-19, it can easily be expanded to include additional variants, vaccination types or doses, and immunity dynamics. Our work highlights the importance of using data-driven modeling methods to guide public health decision-making, particularly in the earlier stages of a pandemic when resources and data may be limited.

## Supporting information

S1 Appendix Model description. Contains additional technical details on model structure, equations, and assumptions, including natural history of disease, transmission, immunity, vaccination, and public health interventions. See Sections A–H for details.
(PDF)

Section A in S1 Appendix Natural history of disease. Describes the stages of SARS-CoV-2 infection (latent, asymptomatic, presymptomatic, symptomatic), their durations, and implications for virus transmissibility in the model.
(PDF)

Section B in S1 Appendix Virus transmission. Details the handling of co-circulating SARS-CoV-2 variants, compartment stratification by variant, and modeling of infection and co-infection dynamics.
(PDF)

Section C in S1 Appendix Immunity to infection. Explains the tracking of immunity and cross-protection against different variants, how susceptibility is updated upon recovery, and the role of cross-variant immunity.
(PDF)

Section D in S1 Appendix Vaccination. Documents the vaccine types modeled, tracking of vaccination status by dose, and the structure for up to three mRNA vaccine doses per individual.
(PDF)

Section E in S1 Appendix Non-pharmaceutical interventions. Outlines interventions such as contact reduction, mask use, tracing, and isolation, and describes their implementation in the model.
(PDF)

Section F in S1 Appendix Model equations. Provides the complete list of model compartments and indices, and presents the system of differential equations governing transmission, vaccination, and interventions.
(PDF)

Section G in S1 Appendix Model calibration. Describes the calibration of epidemiological model parameters using Approximate Bayesian Computation - Sequential Monte Carlo (ABC-SMC), the rationale for this approach, and how ensembles of parameter values were selected to match observed epidemiological data.
(PDF)

Section H in S1 Appendix Software and technical details. C++ code was run on Béluga and Narval HPC clusters; Python 3.10 was used for plotting and visualization.
(PDF)

S1 Table Observed proportion immunized. Proportion of the population that received one or two doses by age group between early 2021 and late 2021.
(PDF)

S1 Fig Demographics of Québec. Measured population size of Québec (left) and the population segmentation considered in the simulation (right).
(TIF)

S2 Fig Hospitalization data. Daily hospitalizations in Québec, stratified by age. Black dots indicate raw daily data, and the dashed lines show 7-day rolling averages.
(TIF)

S3 Fig Calibrated outputs by age. Comparison of observed and model-predicted daily hospitalizations, stratified by age.
(TIF)

S4 Fig Calibration tolerance. Tolerance schedules used in the Approximate Bayesian Computation–Sequential Monte Carlo (ABC-SMC) calibration process.
(TIF)

S5 Figκ valuesPosterior distributions of social-contact reduction coefficients (κ values) estimated during model calibration.(TIF)

S6 Fig Calibrated posteriors. Posterior distributions of key parameters, including baseline probability of transmission, relative infectiousness of asymptomatic cases, isolation probabilities, symptomatic proportions, and age-stratified hospitalization probabilities.
(TIF)

S7 Fig Daily doses. Number of first and second vaccine doses administered to each age group under the simulated prioritization scenarios.
(TIF)

S8 Fig Distribution of doses by age group. Percentage of daily doses allocated to each age group under different prioritization scenarios.
(TIF)

S9 Fig Total doses. Overall percentage of total vaccine doses received by each age group in the simulated prioritization scenarios.
(TIF)

S10 Fig Vaccine coverage, daily hospitalization, and hesitancy. Dynamics of coverage and hospitalizations for nine scenarios (rows) and maximum achievable coverage of 50%, 70%, or 90% (columns).
(TIF)

S11 Fig Daily doses and hesitancy. Time series of daily vaccine doses administered, stratified by age group, for the same nine prioritization scenarios at three different maximum coverage levels.
(TIF)

S12 Fig Total doses and hesitancy. Cumulative doses administered, showing how the lines overlap across scenarios at each coverage level.
(TIF)
