# Supplementary material for: Evaluating COVID-19 vaccination policy in Québec (Canada) using a data-driven dynamic transmission model
Source: PLoS Comput Biol. 2025 Aug 25;21(8):e1013207. doi: 10.1371/journal.pcbi.1013207 (PMC12410880; doi:10.1371/journal.pcbi.1013207)
Supplement: S1 Appendix — Contains additional technical details on model structure, equations, and assumptions, including natural history of disease, transmission, immunity, vaccination, and public health interventions. See Sections A–H for details. (PDF) [file pcbi.1013207.s001.pdf]

Supporting Information – Appendix: Evaluating COVID-19 vaccination policy in Québec (Canada) using a data-driven dynamic transmission model

Samuel Torres-Florez[1], Jorge Luis Flores Anato[2], Jiahuan Helen He[3,4,5], Vicente Garrido Portilla[1,6], Yichen Wu[7], Mathieu Maheu-Giroux[2], Étienne Racine[2,8], Caroline E. Wagner[1]

[1] Department of Bioengineering, McGill University, Montréal, Québec, Canada
[2] Department of Epidemiology and Biostatistics, McGill University, Montréal, Québec, Canada
[3] Department of Physiology, McGill University, Montréal, Québec, Canada
[4] Welch Center for Prevention, Epidemiology, and Clinical Research, Johns Hopkins University, Baltimore, Maryland, USA
[5] Department of Epidemiology, Johns Hopkins Bloomberg School of Public Health, Baltimore, Maryland, USA
[6] Department of Chemical Engineering, University of Waterloo, Waterloo, Ontario, Canada
[7] Department of Electrical and Computer Engineering, McGill University, Montréal, Québec, Canada
[8] Institut national de santé publique du Québec, Québec City, Québec, Canada

Appendix

Dynamic transmission model

Our SARS-CoV-2 transmission model is a deterministic compartmental (SIR-type) dynamic model. The model is designed to capture natural history of disease and immunity, transmission rates, non-pharmacological interventions (NPIs) and vaccination. We describe each component of the model in detail and provide the full model equations at the end.

A Natural history of disease

The natural history of SARS-CoV-2 infection is modeled by a succession of stages that describe symptomatology of disease and virus transmissibility. The first stage is latent infection (E compartments); latent infection is asymptomatic and cannot yet be transmitted to uninfected individuals. The average duration of the latent stage is denoted by $1/\sigma$.

Once the latent stage is over, an infected individual becomes infectious. Upon leaving the latent stage, an individual can take one of two possible pathways: either (i) eventually develop symptoms or (ii) remain asymptomatic for the entire duration of their infection. The proportion of infected individuals who will develop symptoms is defined by w. Individuals who will remain asymptomatic move into the A compartments, while individuals who will develop symptoms move into the P compartments (pre-symptomatic transmissible infection).

Asymptomatic individuals remain infectious until they clear their infection. The average duration of asymptomatic infection is denoted by $1/\gamma^{A,R}$. Upon recovery, asymptomatic infected individuals return to the uninfected compartments (S) and acquire immunity.

The average duration of the pre-symptomatic stage is denoted by $1/\nu$. Individuals that leave the pre-symptomatic stage of infection enter next the symptomatic infectious stage.

Symptomatic infectious individuals belong to I compartments. Upon entering the symptomatic stage of infection, individuals can follow one of two possible pathways: either (i) recover without developing severe symptoms (self-limited infection) or (ii) develop severe symptoms that will require hospitalization. The proportion of symptomatic individuals who will require hospitalization is denoted by y. The average duration of infectiousness after incubation when the infection is self-limited is denoted by $1/\gamma^{I,R}$. When the infection requires hospitalization, the average time between symptom onset (end of incubation) and hospitalization is denoted by $1/\eta^{H}$.

For individuals who are isolated through tracing, the model includes compartments denoted as T to represent those who have been traced and isolated. The T compartments include both asymptomatic and symptomatic individuals who are removed from contact with the community and thus do not contribute to further transmission. Asymptomatic traced individuals move into T^{A}, while symptomatic individuals move into $T^{I,R}$ if they will recover without hospitalization or $T^{I,H}$ if they will require hospitalization. Individuals in T^{A} and $T^{I,R}$ can exit isolation upon recovery, returning to the uninfected (S) compartments, while those in $T^{I,H}$ will proceed to hospitalization. The average duration of isolation in asymptomatic traced individuals is denoted by $1/\gamma^{A,T}$, in symptomatic traced individuals who will recover by $1/\gamma^{I,T}$, and in symptomatic traced individuals who will proceed to hospitalization by $1/\eta^{H,T}$.

Upon hospitalization, individuals can follow one of two possible pathways: either (i) recover from their infection, leave the hospital and return to the pool of uninfected individuals (S compartments) or (ii) die from COVID-19 (D compartments). The proportion of hospitalized COVID-19 patients who will die from their infection is denoted by z. The average duration of hospitalization in those who will recover is denoted by $1/\gamma^{H}$. The average duration of between hospitalization and death is denoted as $1/\mu^{COVID}$. Hospitalized individuals are presumed to be isolated and do not transmit infection, implying that we do not take into account nosocomial infections in our model (these are excluded from the target calibration data). Nosocomial infections were excluded from calibration data.

Initial cases are allocated in the E and I compartments (exposed and infectious individuals) using data for case importation extracted from Godin et al. [1]. We assume that imported cases from travelers are all within the 18-49 and 50-69 age groups, so imported infections are distributed proportionally according to population size in each group.

B Virus transmission

The model can simulate, in principle, any number of SARS-CoV-2 variants circulating simultaneously. Circulating variants are labeled with the index i. Infection by variant i moves an individual from an uninfected compartment S to an infected compartment E_i, representing the latent stage of infection by variant i. We note here that all infected compartments are stratified by the variants causing the infection; therefore all infected compartments will carry a subscript (index) i. Co-infections are not modeled.

Transmission occurs when an uninfected individual comes into contact with a contagious individual. In our model, contacts are stratified with age to reflect social structure. The number of contacts per unit time that an individual in age group a has with individuals of age group a' is defined by the matrix $\Gamma_{aa'}$. Contacts are further stratified by the context in which they occur: in homes without children, in homes with children, at work, at school, in transportation, in leisure activities and in other settings. The complete contact matrix can thus be decomposed in the following sum:

$$\Gamma_{aa'} = \Gamma_{\text{hwc};aa'} + \Gamma_{\text{hwoc};aa'} + \Gamma_{\text{work};aa'} + \Gamma_{\text{sch};aa'} + \Gamma_{\text{trsp};aa'} + \Gamma_{\text{leis};aa'} + \Gamma_{\text{oth};aa'} \tag{1}$$

The values for the components of all contact matrices have been obtained from the CONNECT study [2]. All compartments thus carry an index a denoting age group.

The probability of transmission upon contact between an uninfected individual and an infectious individual depends on three variables: (i) the variant causing infection, (ii) the susceptibility of the uninfected individual to infection (i.e. an immune uninfected individual cannot acquire the infection) and (iii) the presence or absence of symptoms in the infectious individual (asymptomatic individuals are presumed less infectious than symptomatic individuals; pre-symptomatic individuals are presumed as infectious as symptomatic

individuals). The probability of transmission per contact between a fully susceptible individual and a symptomatic individual infected by variant i is denoted by ρ_i^I; if the infected individual is asymptomatic, the probability of transmission is denoted by ρ_i^A. The susceptibility status of an uninfected individual is tracked by the index j, which contains information about susceptibility against all variants. It is defined as follows:

$$j = \sum_{i=0}^{i_{\text{max}}-1} j_i \times 2^{i_{\text{max}}-i-1} \tag{2}$$

where i_{max} is the total number of circulating variants in the model. The value $j_i = 0$ indicates no susceptibility to infection by variant i (immunity to variant i) and a value $j_i = 1$ indicates susceptibility to infection by variant i. The inverse relation is recursive:

$$j_0(j) = \left\lfloor \frac{j}{2^{i_{\text{max}}-1}} \right\rfloor \tag{3}$$

$$j_i(j) = \left\lfloor \frac{j - \sum_{i'=0}^{i-1} j_{i'} \times 2^{i_{\text{max}}-i'-1}}{2^{i_{\text{max}}-i-1}} \right\rfloor \tag{4}$$

All compartments therefore carry an index j to track the susceptibility of individuals to infection by each circulating variant. The probability per unit time that a given uninfected individual in compartment S_{aj} becomes infected by variant i, also called the force of infection due to variant i, is defined by $\lambda_{aj,i}$ and can be written as:

$$\lambda_{aj,i} = \sum_{\bar{a},\bar{j}} \beta^A_{a\bar{a}j\bar{j},i} \frac{A_{\bar{a}\bar{j},i}}{N_{\bar{a}}} + \beta^I_{a\bar{a}j\bar{j},i} \frac{P_{\bar{a}\bar{j},i} + I_{\bar{a}\bar{j},i}}{N_{\bar{a}}} \tag{5}$$

where the transmission tensor components are calculated as:

$$\beta^A_{aa'jj',i} = \Gamma_{aa'} \delta_{j_i(j),0} \delta_{j_i'(j'),1} \rho_i^A,$$
$$\beta^I_{aa'jj',i} = \Gamma_{aa'} \delta_{j_i(j),0} \delta_{j_i'(j'),1} \rho_i^I$$

where δ is the Kronecker delta.

C Immunity to infection

As described in the previous subsection, immunity to infection by any circulating variant i is tracked through the index j defined in Eq.(2).

Whenever an individual recovers from infection by variant i, they gain immunity to future infection by variant i with 100% probability. They may also gain protection against other variants with some probability $< 100\%$; this type of immunity is called cross-protection. Upon recovery from infection by variant i, an individual will move from susceptibility state j' to a new susceptibility state j reflecting acquisition of immunity. Defining $C_{i'i}$ as the probability of acquiring immunity to variant i' following infection by variant i, the probability to recover into state j from state j' following infection by variant i is:

$$p_{\text{rec}}(j|j', i) = \prod_{i'=0}^{i_{\text{max}}-1} \left\{ \delta_{j_{i'}(j'),0} \delta_{j_{i'}(j),0} + \delta_{j_{i'}(j'),1} \left[C_{i'i} \delta_{j_{i'}(j),0} + (1 - C_{i'i}) \delta_{j_{i'}(j),1} \right] \right\} \tag{6}$$

Acquired immunity against infection can wane with time. In our model, waning of immunity is assumed to occur instantaneously (*waning event*). The probability per unit time that a waning event occurs is denoted by χ; conversely, the average duration of acquired immunity against infection is $1/\chi$. Since reinfections were rare prior to Omicron, we assumed than the characteristic timescale for waning for the early variants (wild-type, Alpha and Delta) is on the order of ~ 2 years.

It is further assumed that a waning event wipes immunity against all circulating variants, i.e. there is no selective waning in our model. Therefore, upon a waning event, the probability to wane into state j from state j' is:

$$p_{\text{wan}}(j|j') = \prod_{i=0}^{i_{\text{max}}-1} \delta_{j_i(j),1} \tag{7}$$

Other waning models could be built by modifying Eq.(7).

We do not distinguish between infection- and vaccine-induced immunity against further infection for the purpose of waning.

D Vaccination

We consider only mRNA vaccines in our model, as other types of vaccines have not been widely used in Québec. We do not distinguish between mRNA vaccines from different manufacturers and model them as equivalent. Vaccination status is tracked through an index v, whose value is equal to the number of doses received. The maximum number of doses that an individual can receive over the simulated period is three.

We assume that only uninfected individuals are eligible for vaccination. Since prevalence of active infection remained fairly low prior to Omicron variants, this excludes only a small fraction of individuals. The number of vth vaccine doses administered per unit time as function of time is denoted by $\phi_v(t)$. The number of doses per unit time are calculated dynamically within the model to match real-life vaccine coverage data stratified by age group and number of doses.

D.1 Vaccine effectiveness against infection

Upon vaccination, an individual transitions from v to $v+1$ and their immunity status changes from j' to j, reflecting acquisition of immunity, if any.

The effect of vaccination on an individual's immunity is encoded in a vaccine effectiveness tensor denoted by $\varepsilon_{jj',v}$. The components of this tensor represent the probability that an individual transitions from state j' to state j after receiving their v^{th} dose of vaccine.

To determine the components of the vaccine effectiveness tensor in terms of variant-specific measured vaccine effectiveness of the vth dose against infection $\epsilon_{v,i}$, consider a single variant and the first dose. If, prior to vaccination, the individual is already immune against infection, vaccination will have no effect and keep the individual immune. If, prior to vaccination, the individual is not immune, they will become immune with probability $\epsilon_{v=1,i=0}$ and remain susceptible with probability $1-\epsilon_{v=1,i=0}$. Therefore, for a single variant and the first dose, we obtain:

$$\varepsilon_{jj',v=1} = \delta_{j0}\delta_{j'0} + \delta_{j'1}[\delta_{j0}\epsilon_{v=1,i=0} + \delta_{j1}(1-\epsilon_{v=1,i=0})] \tag{8}$$

or in matrix form:

$$\varepsilon_{jj',v=1} = \begin{bmatrix} 1 & \epsilon_{v=1,i=0} \\ 0 & 1-\epsilon_{v=1,i=0} \end{bmatrix} \tag{9}$$

In the absence of waning, for the second dose, the components of the the vaccine effectiveness tensor are similar to Eq.(12), except that absolute efficacy is replaced by the relative efficacy of the second dose relative to the first dose:

$$\varepsilon_{jj',v=2} = \delta_{j0}\delta_{j'0} + \delta_{j'1}[\delta_{j0}\epsilon^R_{v=2,i=0} + \delta_{j1}(1-\epsilon^R_{v=2,i=0})] \tag{10}$$

where

$$\epsilon^R_{v=2,i=0} = \frac{\epsilon_{v=2,i=0} - \epsilon_{v=1,i=0}}{1-\epsilon_{v=1,i=0}} \tag{11}$$

In general, for the vth dose, in the absence of waning, the components of the vaccine effectiveness tensor are:

$$\varepsilon_{jj',v} = \delta_{j0}\delta_{j'0} + \delta_{j'1}[\delta_{j0}\epsilon^R_{v,i=0} + \delta_{j1}(1 - \epsilon^R_{v,i=0})] \tag{12}$$

where

$$\epsilon^R_{v,i=0} = \frac{\epsilon_{v,i=0} - \epsilon_{v-1,i=0}}{1 - \epsilon_{v-1,i=0}} \tag{13}$$

If there is waning, the relative effectiveness (14) must be modified to account for the probability of having lost immunity in the time interval T_v between dose $v-1$ and dose v:

$$\epsilon^R_{v,i=0} = \frac{\epsilon_{v,i=0} - \exp(-\chi T_v)\epsilon_{v-1,i=0}}{1 - \exp(-\chi T_v)\epsilon_{v-1,i=0}} \tag{14}$$

When there are multiple variants circulating, we generalize Eq.(12) as follows:

$$\varepsilon_{jj',v} = \prod_{i=0}^{i_{\text{max}}-1} \left\{\delta_{j_i(j),0}\delta_{j_i(j'),0} + \delta_{j_i(j'),1}[\delta_{j_i(j),0}\epsilon^R_{v,i} + \delta_{j_i(j),1}(1 - \epsilon^R_{v,i})]\right\} \tag{15}$$

where

$$\epsilon^R_{v,i} = \frac{\epsilon_{v,i} - \exp(-\chi T_v)\epsilon_{v-1,i}}{1 - \exp(-\chi T_v)\epsilon_{v-1,i}} \tag{16}$$

Note that this generalization assumes that upon vaccination, immunity acquisition against each variant is independent, i.e. the probability of acquiring immunity against the Alpha variant is independent of whether or not immunity was acquired against wild-type. We used this approach for simplicity, but other models could be devised by modifying Eq.(15).

D.2 Vaccine effectiveness against hospitalization and death

In our model, protection against infection is tracked through the susceptibility statuses j. However, a direct counterpart for susceptibility to hospitalization is not explicitly incorporated into the model structure. Instead, protection against hospitalization is captured through the probability of being hospitalized given infection. In other words, vaccine-induced immunity against hospitalization and deaths is viewed as a population-level quantity rather than a characteristic of individuals within compartments. This was done for two reasons: (i) to limit the number of compartments and (ii) there was little evidence of waning of immunity against hospitalization.

Since vaccinated individuals who are immune to infection are automatically immune against hospitalization, the probability of hospitalization in vaccinated individuals can be written as:

$$y_v = \left(\frac{1 - \text{efficacy of } v \text{ doses against hospitalization}}{1 - \text{efficacy of } v \text{ doses against infection}}\right) y_{v=0} \tag{17}$$

Similarly, the probability of death in vaccinated can be written as:

$$z_v = \left(\frac{1 - \text{efficacy of } v \text{ doses against death}}{1 - \text{efficacy of } v \text{ doses against hospitalization}}\right) z_{v=0} \tag{18}$$

since, in our model, immunity against hospitalization implies immunity against death.

E Non-pharmaceutical interventions

Two broad categories of non-pharmaceutical interventions were applied in Québec to manage the pandemic in the first two years: (i) reduction of effective contacts through distancing or mask wearing and (ii) contact tracing and isolation of cases.

Reduction of contacts were mostly a consequence of government mandates, for example school and business closures or curfews. We adjusted contacts in our model according to the timeline of mandates during the first two years of the pandemic. Contact reduction was modeled through seven parameters that describe how each type of contact is modified when a given mandate begins or ends:

$$\Gamma_{aa'} = \kappa_{\text{hwc}}\Gamma_{\text{hwc};aa'} + \kappa_{\text{hwoc}}\Gamma_{\text{hwoc};aa'} + \kappa_{\text{work}}\Gamma_{\text{work};aa'} + \kappa_{\text{sch}}\Gamma_{\text{sch};aa'} + \kappa_{\text{trsp}}\Gamma_{\text{trsp};aa'} + \kappa_{\text{leis}}\Gamma_{\text{leis};aa'} + \kappa_{\text{oth}}\Gamma_{\text{oth};aa'} \quad (19)$$

The κ's are scalar coefficients whose values change with time in a step-wise manner and reflect how different types of contacts are affected by various government mandates. The actual values of these coefficients are calibrated but the times at which their values change are not.

Tracing and isolation is modeled by adding a new type of compartment (T) representing individuals who have been traced and isolated; these individuals are not in contact with the community anymore and cannot transmit infection. The proportion of asymptomatic infectious individuals that will be isolated before recovery is denoted by x^A; the proportion of symptomatic infectious individuals that will be isolated before recovery or hospitalization is denoted by x^I. The average time between the end of incubation and isolation in asymptomatics who will be isolated is denoted by $1/\omega^A$; the average time between the end of incubation and isolation in symptomatics who will be isolated is denoted by $1/\omega^I$. An individual may leave a T compartment after recovery or upon hospitalization.

F Model equations

All compartments are stratified according to age a, susceptibility to infection j and vaccination status v; infected compartments are further stratified according to the variant i causing infection. For brevity, the collection of indices a, j and v is shortened using the multi-index $\alpha = \{a, j, v\}$. The list of all compartments and their operational meaning is the following:

$$\begin{aligned}
S_\alpha &= \text{Uninfected individuals}\\
E_{\alpha,i} &= \text{Latent stage of infection}\\
A^R_{\alpha,i} &= \text{Asymptomatics who will recover without being isolated}\\
A^T_{\alpha,i} &= \text{Asymptomatics who will be isolated before recovering}\\
P_{\alpha,i} &= \text{Pre-symptomatic stage of infection}\\
I^R_{\alpha,i} &= \text{Symptomatics who will recover without being isolated or hospitalized}\\
I^H_{\alpha,i} &= \text{Symptomatics who will be hospitalized without being isolated}\\
I^{T,R}_{\alpha,i} &= \text{Symptomatics who will be isolated before recovering, without hospitalization}\\
I^{T,H}_{\alpha,i} &= \text{Symptomatics who will be isolated before hospitalization}\\
T^A_{\alpha,i} &= \text{Asymptomatics who are traced/isolated}\\
T^{I,R}_{\alpha,i} &= \text{Symptomatics who are traced/isolated and will recover without hospitalization}\\
T^{I,H}_{\alpha,i} &= \text{Symptomatics who are traced/isolated and will be hospitalized}\\
H^R_{\alpha,i} &= \text{Hospitalized individuals who will recover}\\
H^D_{\alpha,i} &= \text{Hospitalized individuals who will die from COVID-19}\\
D_{\alpha,i} &= \text{Individuals who have died from COVID-19}
\end{aligned}$$

Some parameters appearing in the model equations have not been defined yet; these are:

$$
\begin{aligned}
b_a &= \text{Number of new individuals per unit time in age group } a \text{ through births and immigration}\\
\mu_a &= \text{Age-specific all-cause mortality rate, excluding COVID-19}\\
\gamma^{A,T}_{\alpha,i} &= \text{Inverse average time asymptomatics spend in isolation before recovering}\\
\gamma^{I,T}_{\alpha,i} &= \text{Inverse average time symptomatics spend in isolation before recovering}\\
\eta^{H,T}_{\alpha,i} &= \text{Inverse average time symptomatics spend in isolation before being hospitalized}
\end{aligned}
$$

The following relations must hold, by definition:

$$
\begin{aligned}
\gamma^{A,T}_{\alpha,i} &= \left(\frac{1}{\gamma^{A,R}_{\alpha,i}} - \frac{1}{\omega^{A}_{\alpha,i}}\right)^{-1}\\
\gamma^{I,T}_{\alpha,i} &= \left(\frac{1}{\gamma^{I,R}_{\alpha,i}} - \frac{1}{\omega^{I}_{\alpha,i}}\right)^{-1}\\
\eta^{H,T}_{\alpha,i} &= \left(\frac{1}{\eta^{H}_{\alpha,i}} - \frac{1}{\omega^{I}_{\alpha,i}}\right)^{-1}
\end{aligned}
$$

The differential equations governing transitions between all compartments are the following:

$$
\begin{aligned}
\dot{E}_{\alpha,i} &= \lambda_{\alpha,i} S_\alpha - (\sigma_{\alpha,i} + \mu_a) E_{\alpha,i}\\
\dot{A}^{R}_{\alpha,i} &= (1 - x^{A}_{\alpha,i})(1 - w_{\alpha,i}) \sigma_{\alpha,i} E_{\alpha,i} - (\gamma^{A,R}_{\alpha,i} + \mu_a) A^{R}_{\alpha,i}\\
\dot{A}^{T}_{\alpha,i} &= x^{A}_{\alpha,i}(1 - w_{\alpha,i}) \sigma_{\alpha,i} E_{\alpha,i} - (\omega^{A}_{\alpha,i} + \mu_a) A^{T}_{\alpha,i}\\
\dot{P}_{\alpha,i} &= w_{\alpha,i} \sigma_{\alpha,i} E_{\alpha,i} - (\nu_{\alpha,i} + \mu_a) P_{\alpha,i},\\
\dot{I}^{R}_{\alpha,i} &= (1 - x^{I}_{\alpha,i})(1 - y_{\alpha,i}) \nu_{\alpha,i} P_{\alpha,i} - (\gamma^{I,R}_{\alpha,i} + \mu_a) I^{R}_{\alpha,i}\\
\dot{I}^{T,R}_{\alpha,i} &= x^{I}_{\alpha,i}(1 - y_{\alpha,i}) \nu_{\alpha,i} P_{\alpha,i} - (\omega^{I}_{\alpha,i} + \mu_a) I^{T,R}_{\alpha,i}\\
\dot{I}^{H}_{\alpha,i} &= (1 - x^{I}_{\alpha,i}) y_{\alpha,i} \nu_{\alpha,i} P_{\alpha,i} - (\eta^{H}_{\alpha,i} + \mu_a) I^{H}_{\alpha,i}\\
\dot{I}^{T,H}_{\alpha,i} &= x^{I}_{\alpha,i} y_{\alpha,i} \nu_{\alpha,i} P_{\alpha,i} - (\omega^{I}_{\alpha,i} + \mu_a) I^{T,H}_{\alpha,i}\\
\dot{T}^{A}_{\alpha,i} &= \omega^{A}_{\alpha,i} A^{T}_{\alpha,i} - (\gamma^{A,T}_{\alpha,i} + \mu_a) T^{A}_{\alpha,i}\\
\dot{T}^{I,R}_{\alpha,i} &= \omega^{I}_{\alpha,i} I^{T,R}_{\alpha,i} - (\gamma^{I,T}_{\alpha,i} + \mu_a) T^{I,R}_{\alpha,i}\\
\dot{T}^{I,H}_{\alpha,i} &= \omega^{I}_{\alpha,i} I^{T,H}_{\alpha,i} - (\eta^{H,T}_{\alpha,i} + \mu_a) T^{I,H}_{\alpha,i}\\
\dot{H}^{R}_{\alpha,i} &= (1 - z_{\alpha,i})(\eta^{H}_{\alpha,i} I^{H}_{\alpha,i} + \eta^{H,T}_{\alpha,i} T^{I,H}_{\alpha,i}) - (\gamma^{H}_{\alpha,i} + \mu_a) H^{R}_{\alpha,i}\\
\dot{H}^{D}_{\alpha,i} &= z_{\alpha,i}(\eta^{H}_{\alpha,i} I^{H}_{\alpha,i} + \eta^{H,T}_{\alpha,i} T^{I,H}_{\alpha,i}) - \mu^{\text{COVID}}_{\alpha,i} H^{D}_{\alpha,i}\\
\dot{D}_{\alpha,i} &= \mu^{\text{COVID}}_{\alpha,i} H^{D}_{\alpha,i}\\
\dot{S}_\alpha &= \dot{S}^{\text{dem.}}_\alpha + \dot{S}^{\text{inf.}}_{\alpha,i} + \dot{S}^{\text{vacc.}}_\alpha + \dot{S}^{\text{rec.}}_\alpha + \dot{S}^{\text{wan.}}_\alpha\\
&= b_a - \mu_a S_\alpha - \lambda_{\alpha,i} S_\alpha + \sum_{\bar{j}} \phi_{a,\bar{j},v} \varepsilon_{a,j,\bar{j};v} - \phi_{a,\bar{j},v}\\
&\quad + \sum_{\bar{\alpha},i} \delta_{a\bar{a}} \delta_{v,\bar{v}} p_{\text{rec}}(j|\bar{j},i)(\gamma^{A,R}_{\bar{\alpha},i} A^{R}_{\bar{\alpha},i} + \gamma^{A,T}_{\bar{\alpha},i} T^{A}_{\bar{\alpha},i} + \gamma^{I,R}_{\bar{\alpha},i} I^{R}_{\bar{\alpha},i} + \gamma^{I,T}_{\bar{\alpha},i} T^{I,R}_{\bar{\alpha},i} + \gamma^{H}_{\bar{\alpha},i} H^{R}_{\bar{\alpha},i})\\
&\quad + \sum_{j} p_{\text{wan}}(j|\bar{j}) \chi_{a,\bar{j},v} - \sum_{j} p_{\text{wan}}(j|\bar{j}) \chi_\alpha S_\alpha
\end{aligned}
$$

G Model calibration

G.1 Calibration process introduction

The calibration of epidemiological models is a crucial step in ensuring their predictive reliability and accuracy. The goal of model calibration is to identify ensembles of model parameter values for which model output accurately reproduces specific sets of observed data (epidemiological targets). In this work, we employed the Approximate Bayesian Computation - Sequential Monte Carlo (ABC-SMC) method for calibration. This choice was motivated by the ABC-SMC method's efficacy in handling complex models with numerous parameters, which are not easily tractable with traditional likelihood-based approaches.

The ABC-SMC method is an iterative process where parameter value estimates are progressively refined by comparing model output to observed data, with increasingly strict acceptance criteria. The list of calibrated parameter values and the calibration algorithm itself are detailed below. Epidemiological target data for calibration were daily new hospitalizations per age group and proportion of cases attributed to each circulating variant. The model was calibrated for 20 iterations using 100 particles (i.e., parameter sets; see below).

G.2 Calibration methodology

Algorithm structure

An ensemble of model parameter values is called a "particle" and is represented by a vector $\boldsymbol{\theta}$. The objective of the calibration algorithm is to find particles for which model output fits observed data "well enough". This set of particles constitutes a sample of an approximate posterior distribution for model parameter values.

In a nutshell, the calibration algorithm is structured as follows. The first iteration of the ABC-SMC scheme consists of the following steps:

1. Sample a particle from prior distribution
2. Run the model with the sampled particle
3. Compare model output with epidemiological target data
4. If model output fits data "well enough", retain the particle as an acceptable candidate
5. If model output does not fit well enough, reject the particle
6. Repeat the above steps until a given number of particles have been accepted

The collection of accepted particles is referred to as a "population". Once the first population is created, the algorithm proceeds as follows:

1. Sample a particle from the last population of accepted particles
2. Slightly modify parameter values of the sampled particle ("particle perturbation")
3. Run the model with the perturbed particle
4. Compare model output with epidemiological target data
5. If model output fits data "well enough", retain the perturbed particle as an acceptable candidate
6. If model output does not fit well enough, reject the perturbed particle
7. Repeat the above steps until a given number of particles have been accepted

At the end of this sequence of steps, a new population of accepted particles is generated; this constitutes an "iteration" of the algorithm. Iterations of the algorithm are labeled with the index q. Each subsequent iteration produces a different population because the criteria that define what is meant by "fitting the data well enough" become more and more strict.

The spirit of the algorithm is to start with relatively loose criteria to define a "good enough fit" and refine these criteria progressively to obtain better and better fits.

Parameter selection

Parameters were chosen for calibration based on their influence on model dynamics and their level of uncertainty or variability in the literature. The parameter values that we calibrated with ABC-SMC are:

(i) The baseline probabilities of transmission per infectious contact per variant
(ii) The relative probability of transmission in asymptomatic individuals per variant
(iii) The proportion of infected individuals that will be hospitalized per age group
(iv) The proportion of infected individuals that will develop symptoms
(v) The proportion of infectious individuals that will be isolated, stratified by presence/absence of symptoms
(vi) The seeding of Alpha and Delta variants (cases)
(vii) The number of contacts per unit time through the values of the κ coefficients

Distance tolerance and weights

Comparison between model output and epidemiological target data is performed using a metric called the "distance function". This distance function, denoted by $d(\boldsymbol{\theta})$, quantifies the discrepancy between model outputs generated with particle $\boldsymbol{\theta}$ and observed data. The choice of this distance function is arbitrary.

We employed a mixed distance measure, incorporating multiple data streams such as hospitalization incidence and the proportion of cases attributed to the Alpha and delta variants, to capture the model's fidelity across different targets. For a given particle $\boldsymbol{\theta}$, its associated model prediction $\hat{Y}_{\boldsymbol{\theta}}(t)$, and the observed data $Y(t)$, our distance function is defined as:

$$d(\boldsymbol{\theta}) \equiv \sum_t \frac{[\hat{Y}_{\boldsymbol{\theta}}(t) - Y(t)]^2}{Y(t)}$$

This distance function is essentially a chi-squared type of metric. The sum over t means summing over all daily values (the model time step is one day).

A particle is accepted, i.e. considered a viable candidate for being part of a representative sample of the posterior distribution, if its distance is less or equal than some tolerance threshold. This tolerance threshold defines what is meant by "fitting the data well enough". At initial iteration $q = 0$, the tolerance thresholds ϵ_0 remain fairly loose, allowing for easy acceptance of particles. The thresholds ϵ_q (not to be confused with vaccine effectiveness) are gradually tightened in subsequent iterations such that the quality of the fit increases, yielding refined parameter value estimates. The acceptance of a particle $\boldsymbol{\theta}^*$ during iteration q is conditional on $d(\boldsymbol{\theta}^*) \leq \epsilon_q$. The choice of a sequence of tolerance thresholds ϵ_q is arbitrary and is guided by the efficiency of the algorithm. Our final choice for a sequence of thresholds ϵ_q was obtained by trial and error.

Each accepted particle $\boldsymbol{\theta}^*$ is assigned a weight $w(\boldsymbol{\theta}^*)$, which is inversely proportional to its distance from the observed data:

$$w(\theta^*) \propto \frac{1}{d(\theta^*)^2}$$

Particles weights are used when sampling from the last population of accepted particles. These weights are normalized to sum to unity.

G.3 Parameter priors

The priors were beta distributions whose mode was determined by calibrating the model by hand and based in measured values from the literature. The variance of each beta distribution priors was determined by trial and error to allow a good enough acceptance rate for the first population.

G.4 Calibration procedure

Initial setup

Calibration with ABC-SMC requires prior distributions for each parameter, reflecting initial knowledge about their plausible values based on literature review and expert opinion. The initial tolerance threshold ϵ_0 was set to a value ensuring a broad acceptance of particles, facilitating exploration of the parameter space.

Detailed iterative process

1. Particle generation: At the initial iteration $q = 0$, particles are drawn from the prior distributions. For subsequent iterations $q > 0$, particles are selected from the accepted set θ^* of the previous iteration $q - 1$, with selection probability equal to their weight w_{q-1}.
2. Perturbation: For iterations $q > 0$, sampled particles (parameter values) $\boldsymbol{\theta}^*$ were slightly modified to explore parameter space in their neighborhood. Modification of parameter values is called "perturbation". Perturbation is crucial because this is how better fits to target data are identified. Perturbation was achieved by sampling a new particle from a multidimensional beta distribution whose mode is $\boldsymbol{\theta}^*$ and whose variance is pre-determined. In the first few iterations, the variance of the perturbation is larger to allow wider exploration of parameter space. As q increases, the variance of the perturbation decreases since finer exploration of parameter space is required. The perturbed particle is denoted as $\boldsymbol{\theta}^{**}$.
3. Model simulation: For each particle $\boldsymbol{\theta}^{**}$, the model is executed, and outputs are generated and compared with observed data.
4. Distance calculation: The distance $d(\theta^{**})$ between model outputs and observed data was computed using the metric described previously.
5. Particle acceptance: A particle $\boldsymbol{\theta}^{**}$ was accepted during iteration q if $d(\boldsymbol{\theta}^{**}) \leq \epsilon_q$. Upon acceptance in population q, a particle is attributed a weight that is inversely proportional to its distance.
6. Tolerance adjustment: The tolerance threshold ϵ_q is reduced at each iteration to tighten the acceptance criteria. This allows for the identification of particles that provide increasingly better fits to the observed data.

After the last iteration is complete, the population of accepted particles constitutes an approximate sample of the posterior distribution that would have been obtained from a likelihood-based method.

H Software and technical details

The source code is written in C++, and the simulations and calibrations were executed using multi-thread programming on high-performance computing (HPC) systems. The HPC resources were provided by the Digital Research Alliance of Canada through their Béluga and Narval clusters. Plotting and visualization were performed using Python 3.10.

References

[1] Godin A, Xia Y, Buckeridge DL, Mishra S, Douwes-Schultz D, Shen Y, et al. The role of case importation in explaining differences in early SARS-CoV-2 transmission dynamics in Canada—A mathematical modeling study of surveillance data. International Journal of Infectious Diseases. 2021;102:254–259.

[2] Drolet M, Godbout A, Mondor M, Béraud G, Drolet-Roy L, Lemieux-Mellouki P, et al. Time trends in social contacts before and during the COVID-19 pandemic: the CONNECT study. BMC Public Health. 2022;22(1):1–12.
