# Supplementary material for: Evaluating COVID-19 vaccination policy in Québec (Canada) using a data-driven dynamic transmission model
Source: PLoS Comput Biol. 2025 Aug 25;21(8):e1013207. doi: 10.1371/journal.pcbi.1013207 (PMC12410880; doi:10.1371/journal.pcbi.1013207)
Supplement: S1 Table — Proportion of the population that received one or two doses by age group between early 2021 and late 2021. (PDF) [file pcbi.1013207.s010.pdf]

Supplementary Table 1: Observed proportion immunized. Proportion of the population that have received one or two doses by age group between early 2021 and late 2021.

**Dose**	**0-4**	**5-11**	**12-17**	**18-49**	**50-69**	**70+**
Percentage 1st dose	0.00	0.00	0.86	0.85	0.93	0.86
Percentage 2nd dose	0.00	0.00	0.79	0.80	0.91	0.84
