# Supplementary figures and images for: Evaluating COVID-19 vaccination policy in Québec (Canada) using a data-driven dynamic transmission model
Source: PLoS Comput Biol. 2025 Aug 25;21(8):e1013207. doi: 10.1371/journal.pcbi.1013207 (PMC12410880; doi:10.1371/journal.pcbi.1013207)

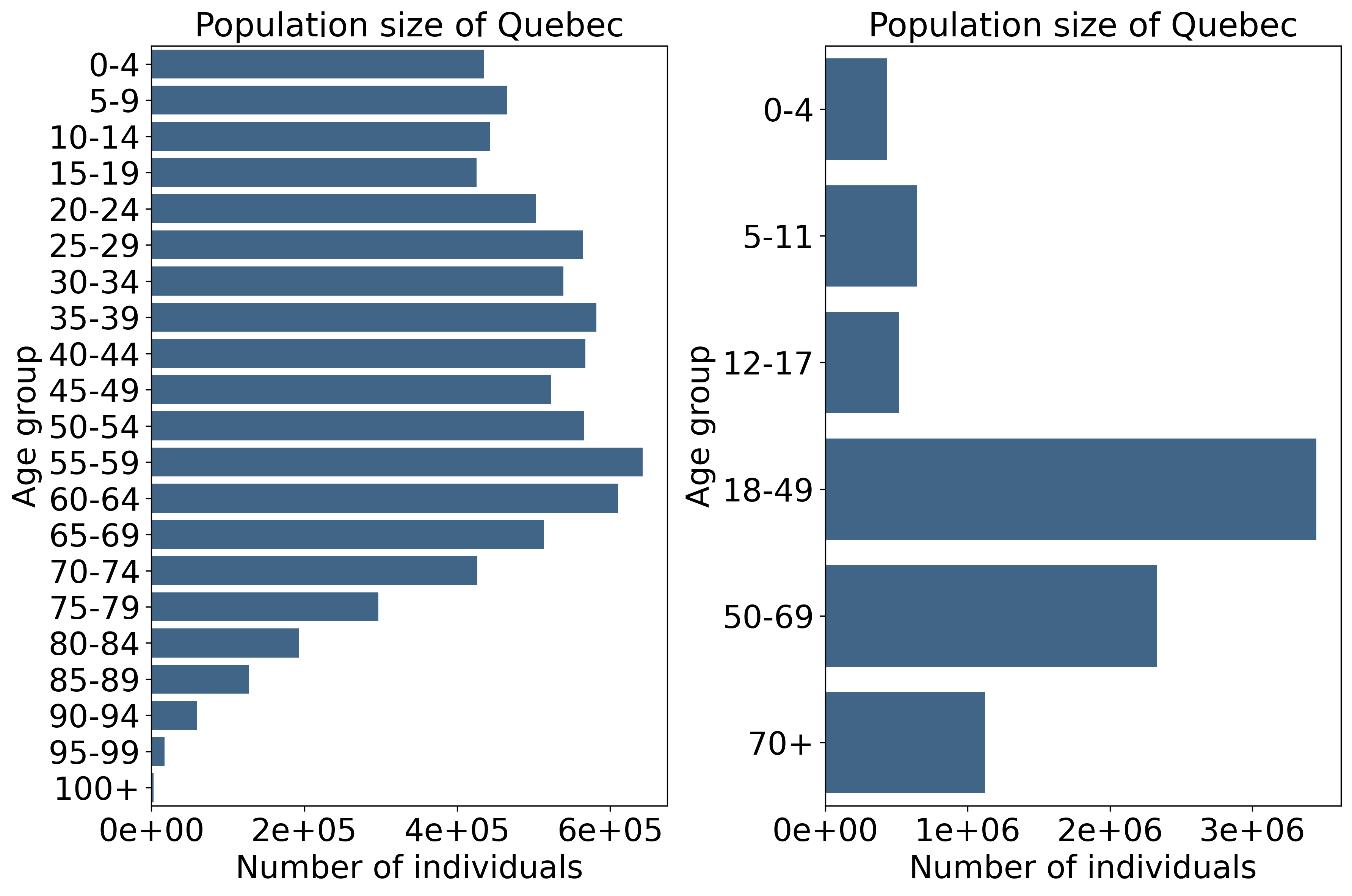

Supplement: S1 Fig — Measured population size of Québec (left) and the population segmentation considered in the simulation (right). (TIF) [file pcbi.1013207.s011.tiff]

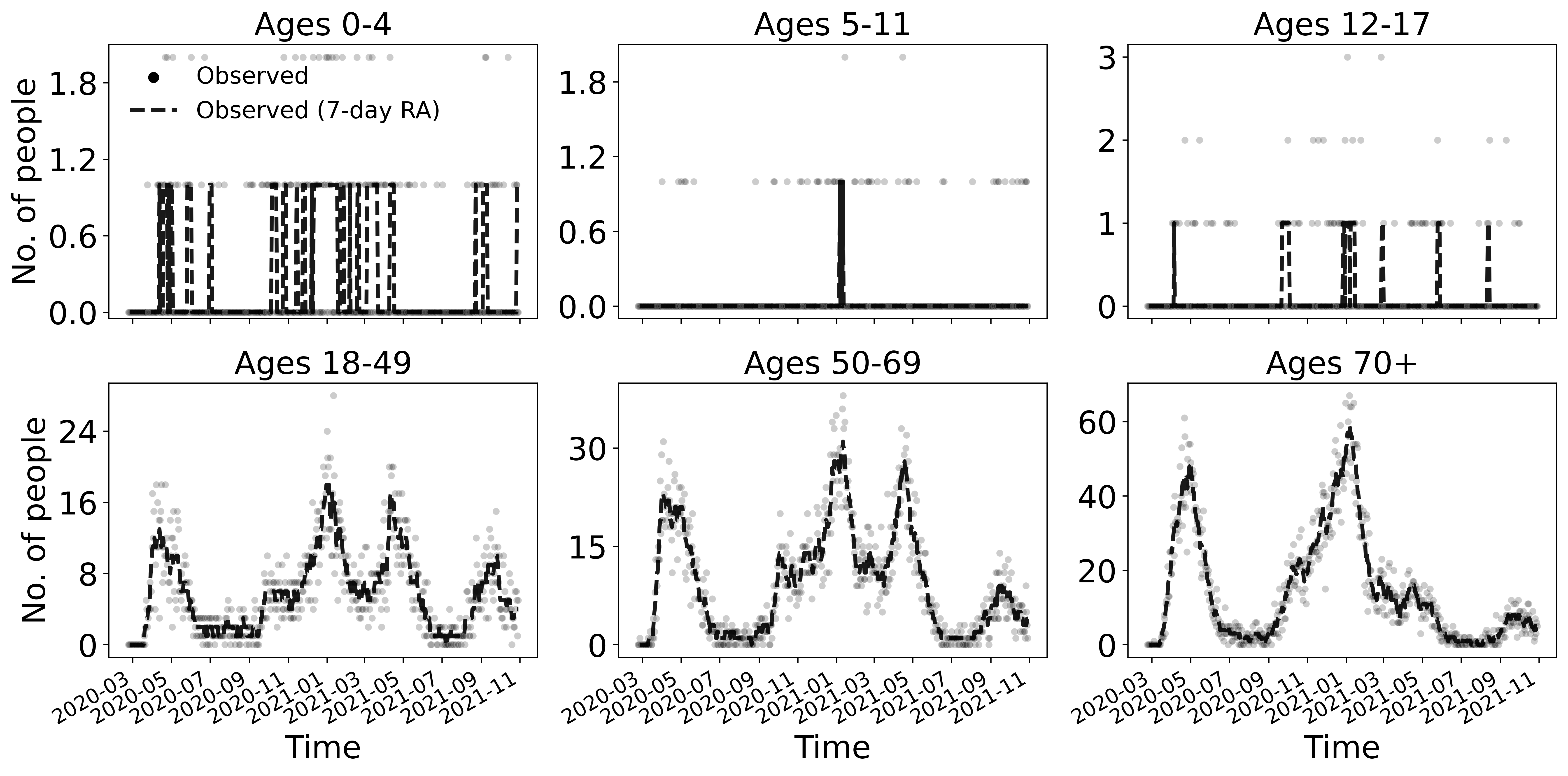

Supplement: S2 Fig — Daily hospitalizations in Québec, stratified by age. Black dots indicate raw daily data, and the dashed lines show 7-day rolling averages. (TIF) [file pcbi.1013207.s012.tiff]

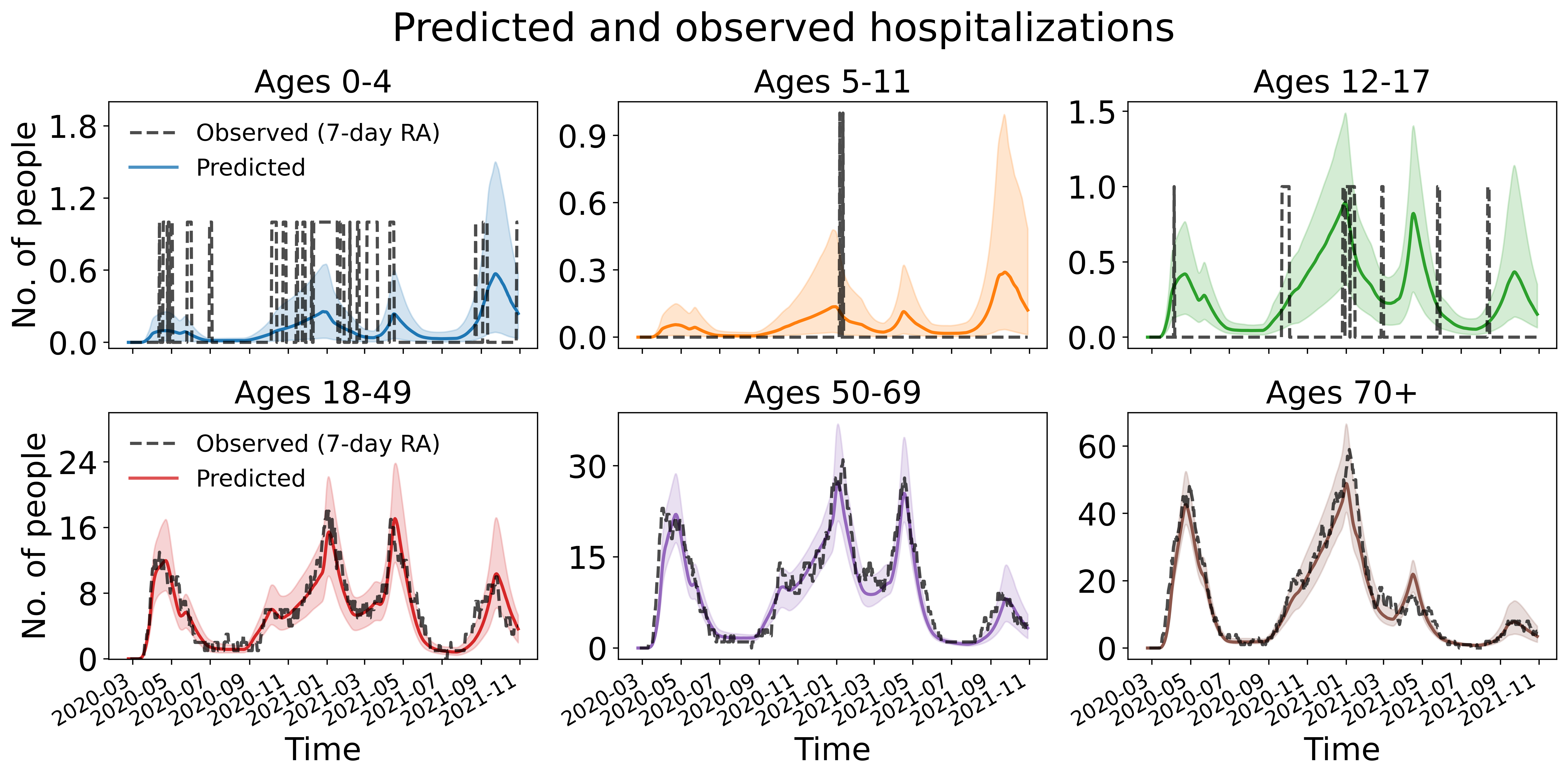

Supplement: S3 Fig — Comparison of observed and model-predicted daily hospitalizations, stratified by age. (TIF) [file pcbi.1013207.s013.tiff]

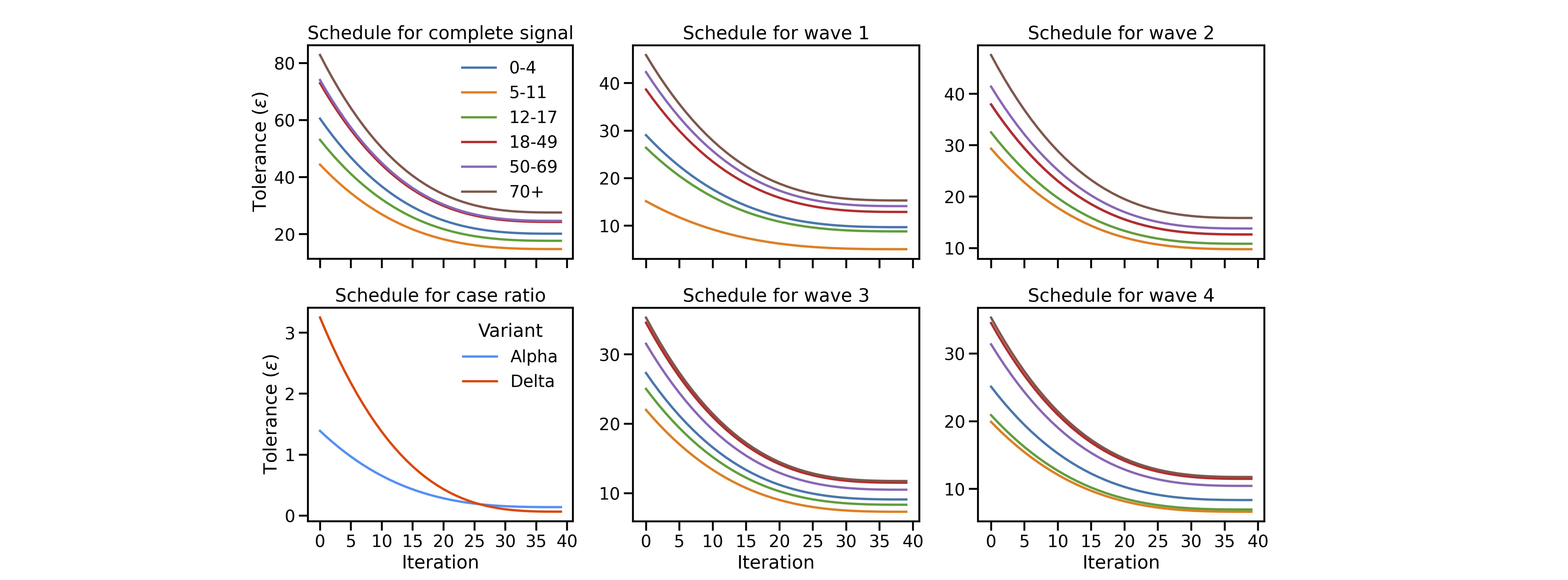

Supplement: S4 Fig — Tolerance schedules used in the Approximate Bayesian Computation–Sequential Monte Carlo (ABC-SMC) calibration process. (TIF) [file pcbi.1013207.s014.tiff]

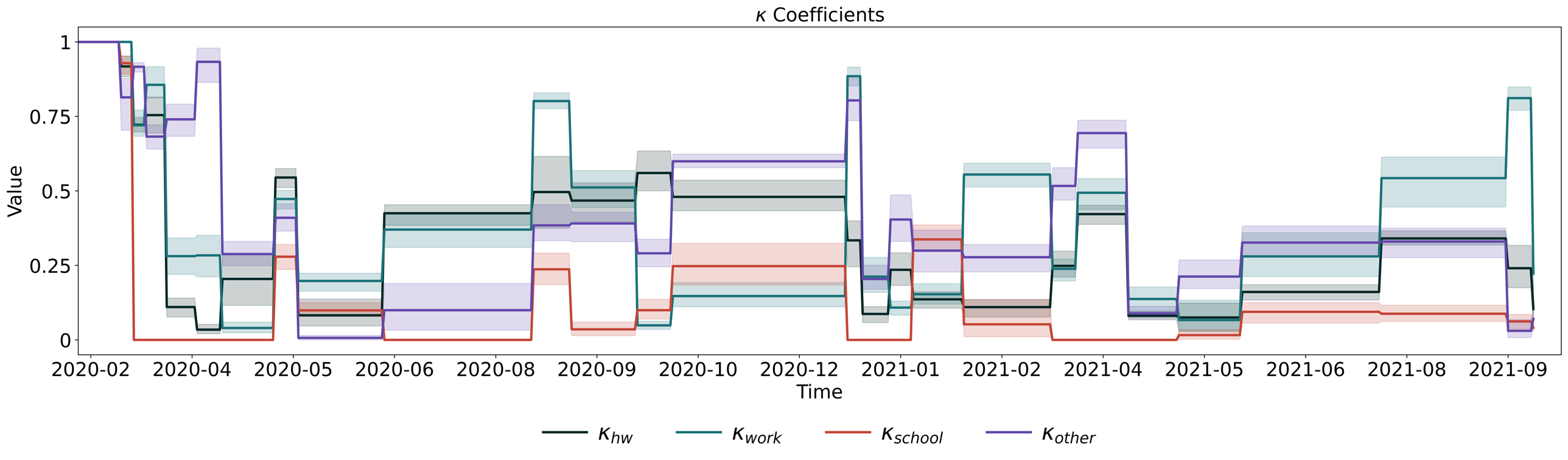

Supplement: S5 Fig — Posterior distributions of social-contact reduction coefficients (κ values) estimated during model calibration. (TIF) [file pcbi.1013207.s015.tiff]

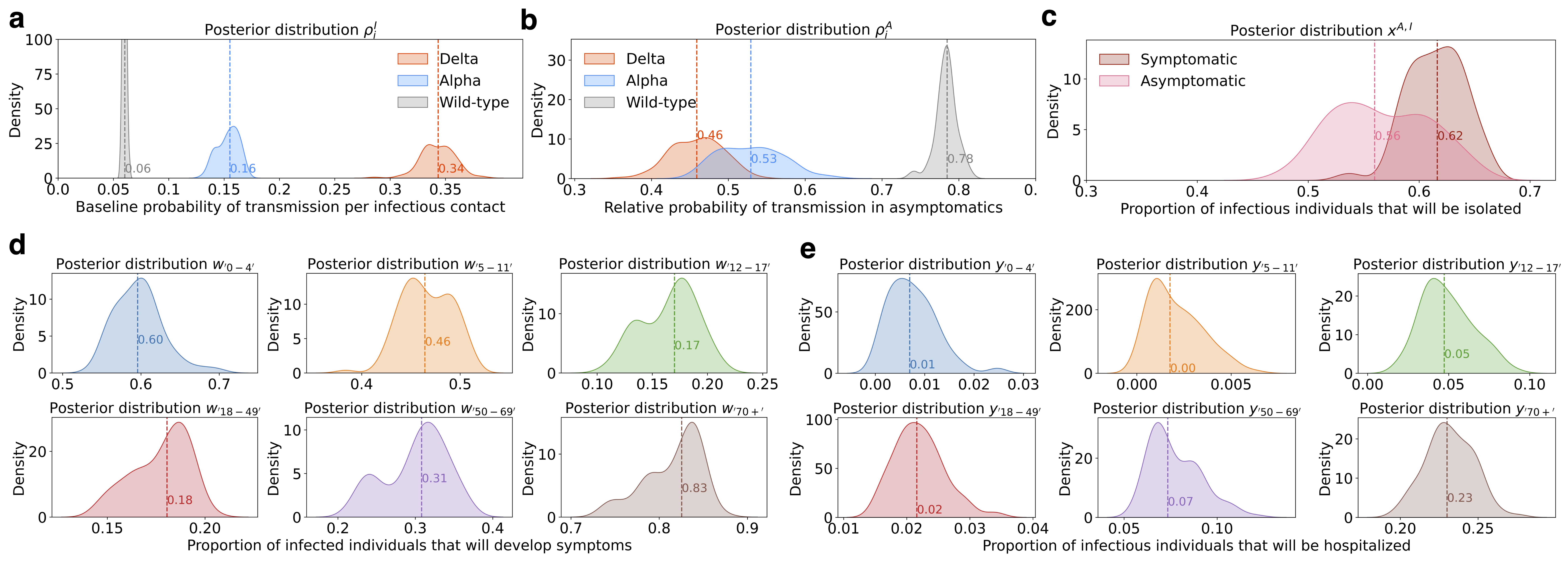

Supplement: S6 Fig — Posterior distributions of key parameters, including baseline probability of transmission, relative infectiousness of asymptomatic cases, isolation probabilities, symptomatic proportions, and age-stratified hospitalization probabilities. (TIF) [file pcbi.1013207.s016.tiff]

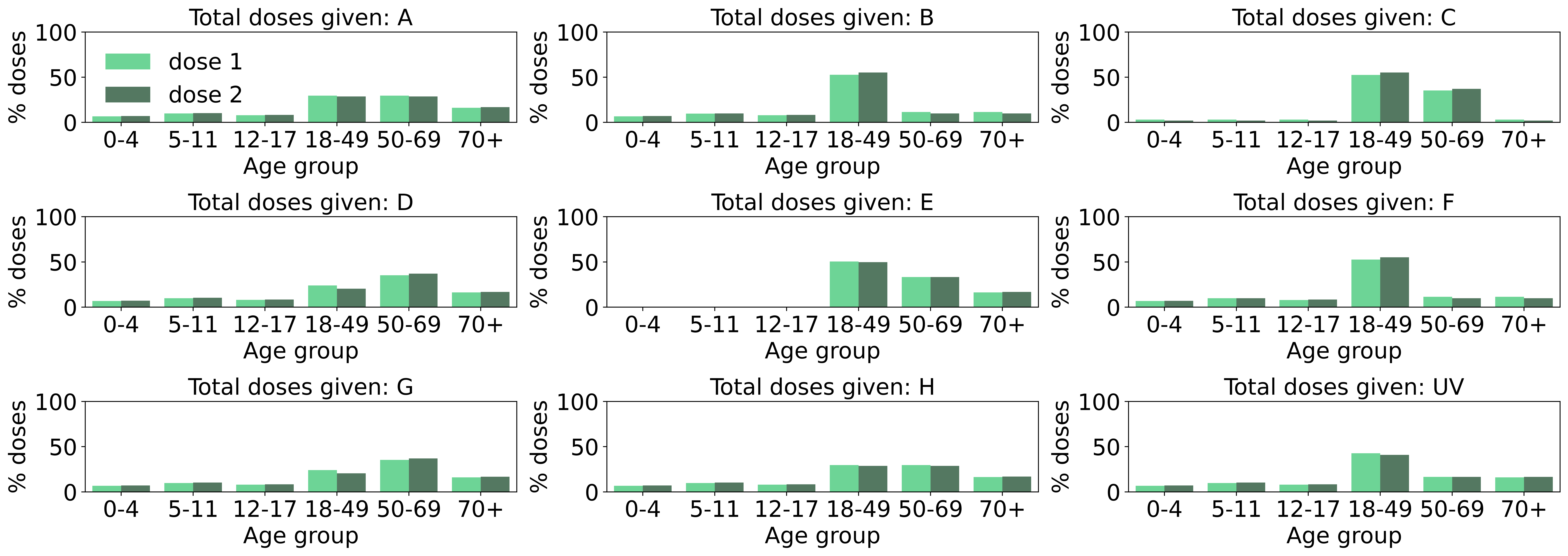

Supplement: S9 Fig — Overall percentage of total vaccine doses received by each age group in the simulated prioritization scenarios. (TIF) [file pcbi.1013207.s019.tiff]

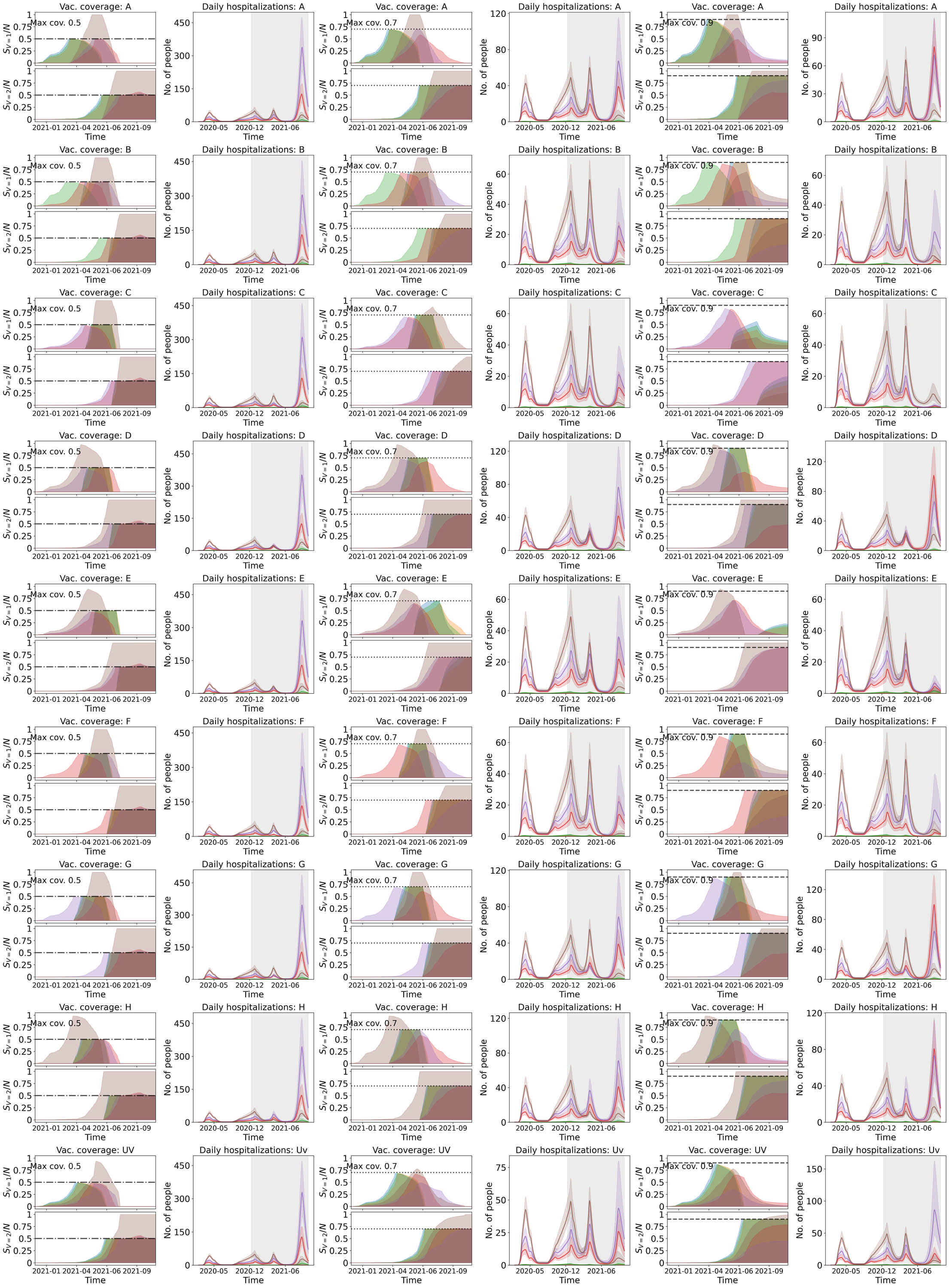

Supplement: S10 Fig — Dynamics of coverage and hospitalizations for nine scenarios (rows) and maximum achievable coverage of 50%, 70%, or 90% (columns). (TIF) [file pcbi.1013207.s020.tiff]

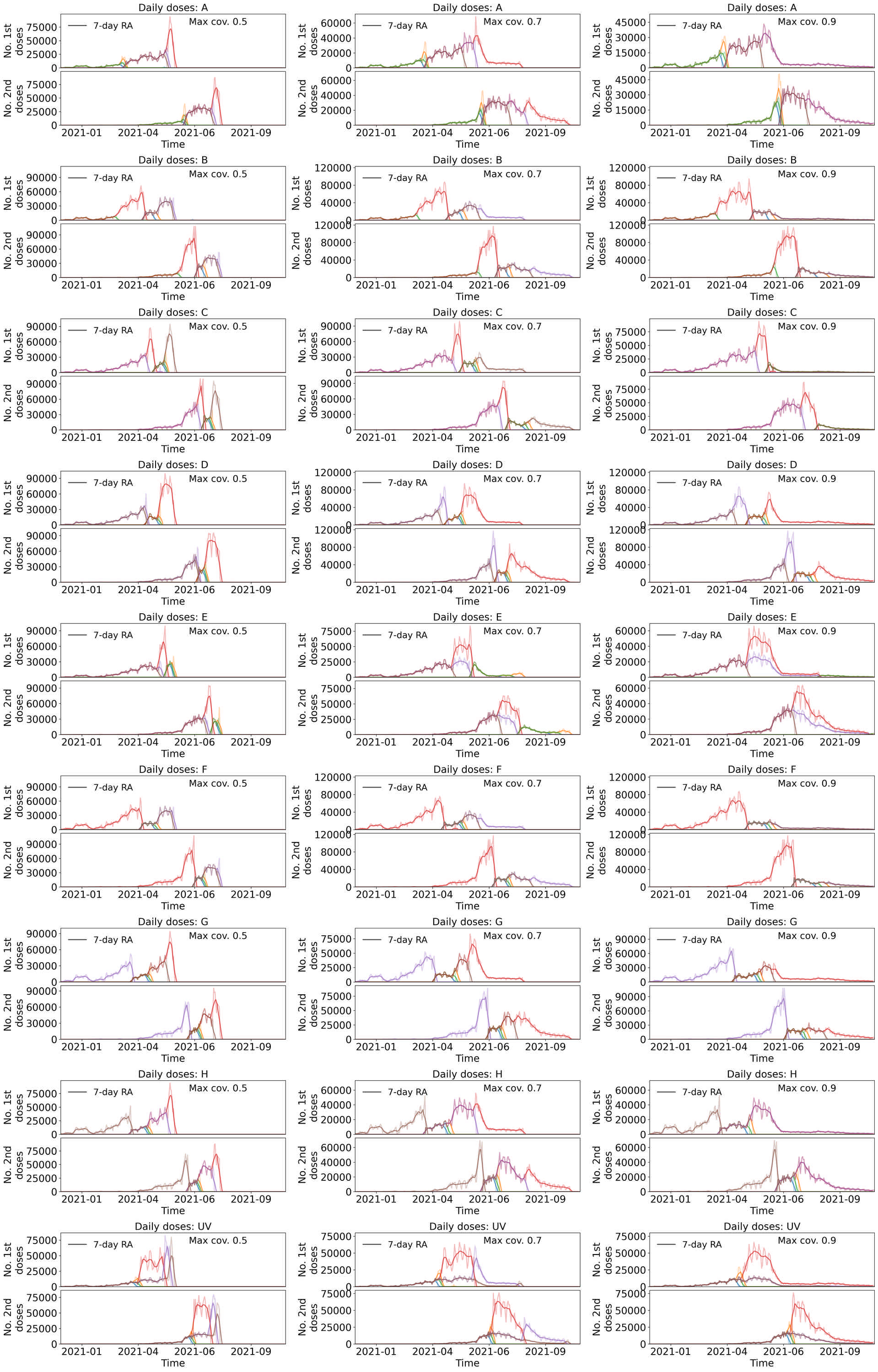

Supplement: S11 Fig — Time series of daily vaccine doses administered, stratified by age group, for the same nine prioritization scenarios at three different maximum coverage levels. (TIF) [file pcbi.1013207.s021.tiff]

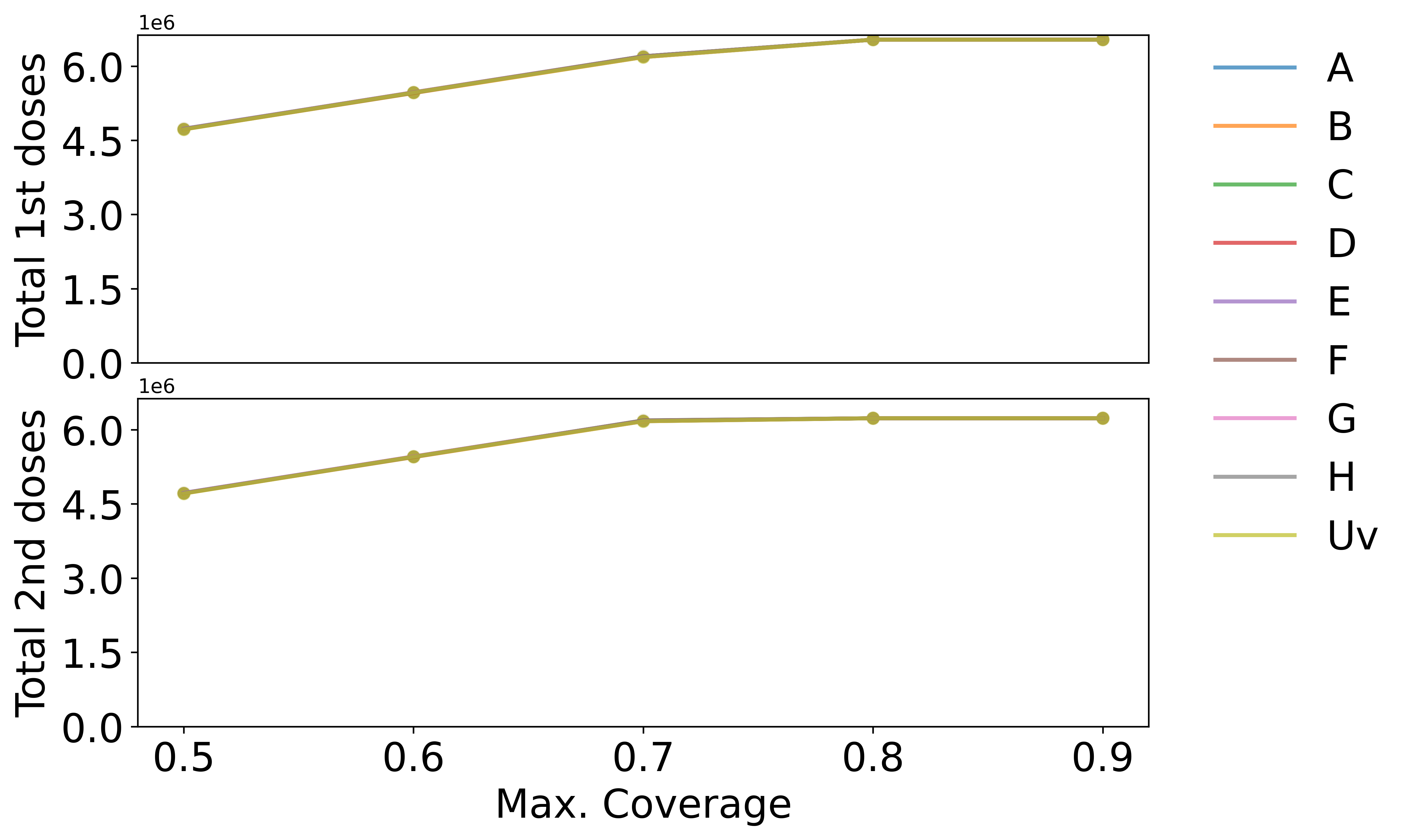

Supplement: S12 Fig — Cumulative doses administered, showing how the lines overlap across scenarios at each coverage level. (TIF) [file pcbi.1013207.s022.tiff]
